# Cross-fitted instrument: A blueprint for one-sample Mendelian randomization

**DOI:** 10.1371/journal.pcbi.1010268

**Published:** 2022-08-29

**Authors:** William R. P. Denault, Jon Bohlin, Christian M. Page, Stephen Burgess, Astanand Jugessur

**Affiliations:** 1 Department of Human Genetics, University of Chicago, Chicago, Illinois, United States of America; 2 Centre for Fertility and Health, Norwegian Institute of Public Health, Oslo, Norway; 3 Department of Method Development and Analytics, Norwegian Institute of Public Health, Oslo, Norway; 4 Department of Mathematics, Faculty of Mathematics and Natural Sciences, University of Oslo, Oslo, Norway; 5 MRC Biostatistics Unit, School of Clinical Medicine, University of Cambridge, Cambridge, United Kingdom; 6 Department of Global Public Health and Primary Care, University of Bergen, Bergen, Norway; Université de Montréal, CANADA

## Abstract

Bias from weak instruments may undermine the ability to estimate causal effects in instrumental variable regression (IVR). We present here a new approach to handling weak instrument bias through the application of a new type of instrumental variable coined ‘Cross-Fitted Instrument’ (CFI). CFI splits the data at random and estimates the impact of the instrument on the exposure in each partition. These estimates are then used to perform an IVR on each partition. We adapt CFI to the Mendelian randomization (MR) setting and term this adaptation ‘Cross-Fitting for Mendelian Randomization’ (CFMR). We show that, even when using weak instruments, CFMR is, at worst, biased towards the null, which makes it a conservative one-sample MR approach. In particular, CFMR remains conservative even when the two samples used to perform the MR analysis completely overlap, whereas current state-of-the-art approaches (e.g., MR RAPS) display substantial bias in this setting. Another major advantage of CFMR lies in its use of all of the available data to select genetic instruments, which maximizes statistical power, as opposed to traditional two-sample MR where only part of the data is used to select the instrument. Consequently, CFMR is able to enhance statistical power in consortia-led meta-analyses by enabling a conservative one-sample MR to be performed in each cohort prior to a meta-analysis of the results across all the cohorts. In addition, CFMR enables a cross-ethnic MR analysis by accounting for ethnic heterogeneity, which is particularly important in meta-analyses where the participating cohorts may have different ethnicities. To our knowledge, none of the current MR approaches can account for such heterogeneity. Finally, CFMR enables the application of MR to exposures that are either rare or difficult to measure, which would normally preclude their analysis in the regular two-sample MR setting.

## Author summary

We present a new approach to handling weak instrument bias through the use of a new type of instrumental variable that enables a conservative one-sample Mendelian Randomization. The new method provides the same power as the standard two-sample Mendelian Randomization but does not require summary statistics from a previously published genome-wide association study in an independent cohort to build the instrument. In particular, our method can quantify the effect of exposures that are either rare or difficult to measure, which is almost unfeasible with current Mendelian Randomization methods. Finally, our approach enables a cross-ethnic instrumental variable regression to account for heterogeneity in a multi-ethnic sample and is also well-adapted to a meta-analysis setting whereby summary statistics from many participating cohorts are analyzed jointly.

This is a *PLOS Computational Biology* Methods paper.

## 1 Introduction

Mendelian Randomization (MR) is the most widely used bioinformatics tool for causal inference. By exploiting the random segregation of alleles during meiosis, MR enables testing for the causal effect of a modifiable exposure on an outcome of interest with minimal risk of confounding. Two-sample MR is considered the gold standard of MR methods [1] because it has higher power than other MR methods and guarantees conservative estimates even in the presence of weak instruments [2, 3]. Two-sample MR relies on two key assumptions regarding the sample used to validate the instrument and the sample used to estimate the causal effect:

- The instrument (‘gene-exposure’) and the causal effect (‘exposure-outcome’) have to be estimated in non-overlapping samples, otherwise the estimates would be biased toward the confounding effect [4, 5].
- The two samples have to derive from a similar population, i.e., they must have a similar age distribution, sex ratio, and ethnicity, among others. As with the first assumption, bias is expected if this assumption is violated [6].

When performing a two-sample MR, the first sample is used to select genetic instruments that are robustly associated with the exposure and the second sample is used to estimate the causal effect of the exposure on the outcome using the genetic instruments selected in the first sample. In most two-sample MR settings, the lead single-nucleotide polymorphisms (SNPs) identified from a large GWAS or genome-wide association meta-analysis (GWAMA) that show robust associations with the exposure are routinely used to build the genetic instrument to be used in the first sample [7]. Thus, performing a two-sample MR using an exposure for which there are no previously reported summary statistics requires coordinating the analysis between at least two large cohorts from a similar population, which is time-consuming and often unfeasible.

One-sample MR can be used when two-sample MR is not feasible. Compared to two-sample MR, one-sample MR employs the same sample to identify genetic variants as instruments for estimating the effect of interest. However, one-sample MR approaches are heavily influenced by weak instrument bias and the winner’s curse, which can be substantial in finite samples [8]. We refer to these two sources of bias as ‘endogeneity bias’ in the rest of the article. Further, if not stated otherwise, we refer to ‘one-sample MR’ as one-sample MR methods that do not account for endogeneity bias. One-sample MR approaches can have severely inflated type I error and produce spurious findings [8] when the endogeneity bias shifts the estimate of the causal effect toward the confounded effect [5, 9], as opposed to two-sample MR in which the estimate is simply biased toward the null.

Here, we present a novel approach to performing one-sample MR that accounts for endogeneity bias and adequately controls the type I error even under extreme scenarios where the instruments are very weak (e.g., with an explained variance of ≤0.01%) and the association is strongly confounded. Under such scenarios, the standard one-sample MR is heavily biased and has an inflated type I error. Our solution relies on a simple modification of the two-stage least square (2SLS) procedure [10] that satisfies the main assumptions of two-sample MR (i.e., the samples are non-overlapping and stem from a similar population) while using only a single dataset for instrument selection. This modification exploits the concept of cross-fitting (CF) from the double/debiased machine-learning (DML) approach proposed by Chernozhukov *et al.* [11]. In essence, we construct a new type of instrument based on CF, coined ‘Cross-Fitted Instrument’ (CFI), that allows a conservative estimation of the causal effect of an exposure on a given outcome. In the rest of the article, we refer to a conservative estimate as one which is, at worst, biased toward zero. Other ideas analogous to CF can be found in the earlier works by Angrist *et al.* [12, 13]. CFI differs from these approaches in that it uses a data-splitting procedure that allows all of the available data to be used in instrument selection, thus eliminating the need for sub-sampling [12]. Consequently, CFI is able to reduce the computational burden compared to the jackknife procedure by Angrist *et al.* [13].

We term the adaption of CFI to MR as ‘Cross-Fitting for Mendelian Randomization’ (CFMR) and show that it exploits more of the available data than traditional two-sample MR when estimating the causal effect of an exposure on an outcome of interest. Other works on debiased one-sample MR have recently appeared in the literature [8, 14, 15], but the need for a sufficiently large sample size [14] or being restricted to inverse-variance two-sample weighting MR [8, 15] are recognized limitations. By contrast, CFMR is applicable to smaller sample sizes and is easily adaptable to a polygenic risk score (PRS) setting [16]. Finally, our work is also related to the recently proposed ‘causal gradient boosting’ approach by Bakhitov and Singh [17]. Like us, Bakhitov and Singh [17] also use CF to construct a new type of instrument. Specifically, they use CF to build an aggregated predictor of the exposure. CFMR differs from their approach in that it uses CF to build an instrument using different predictors of the exposure to generate its components.

## 2 Methods

In their pioneering work, Chernozhukov *et al.* [11] proposed two causal estimators, DML1 and DML2, that are asymptotically equivalent. We present here their MR counterparts, CFMR1 and CFMR2, that are also asymptotically equivalent. For extensive details regarding the optimal selection of genetic instruments, readers are referred to the work by Hemani *et al.* [16]. Here, we restrict ourselves to the case where the genetic instrument does not exhibit pleiotropic effects [18]. In the subsections below, we explain the concept of *K*-fold CFI and define the estimators CFMR1 and CFMR2. To further ease comprehension, we also provide a simple example of the 2-fold CFMR in the S1 Appendix (see the subsection called ‘2-fold CFMR’).

### 2.1 Setup

Let *Y* be a continuous outcome, *X* a continuous exposure, and *Z* a matrix containing Υ instruments. We assume that *Y*, *X* and *Z* are connected through the following linear regression models:

$$\begin{array}{c} Y=\beta_{0}X+U,\;E\left[U|\Pi ,Z\right]=0 \end{array}$$ (1)

$$\begin{array}{c} X=Z\Pi +V,\;E[V|Z]=0 \end{array}$$ (2)

The parameter of interest, *β*_0_, is the causal effect of X on Y, Π is the vector of regression coefficients for the instruments, *U* and *V* are two correlated errors, and E is the expectation operator. For the sake of simplicity, we focus on a linear relationship between the instruments (*Z*) and the exposure (*X*). However, our approach can easily handle non-linear relationships between *Z* and *X* (see the section below).

#### 2.1.1 *K*-fold CFI

A CFI based on *K* ≥ 2 splits is referred to as a *K*-fold CFI, which can be described as follows. Let us consider *K*-fold random partitions of the observation indices [*N*] = (1, …, *N*), where the size of each fold is $\frac{N}{K}$. We refer to these partitions as (*I*_*k*_)_*k*∈1: *K*_. For each *k* ∈ (1, …, *K*), we define the complement of the partition *I*_*k*_ as $I_{k}^{c}=\left\{1,\ldots .,N\notin I_{k}\right\}$. For each *k*, we select Υ_*k*_ independent variants ${\tilde{Z}}_{k}=\left(Z_{1,k},\ldots ,Z_{\Upsilon_{k},k}\right)$ by performing a GWAS of the continuous exposure *X* using the data in $I_{k}^{c}$. In our application to a real dataset (see the subsection ‘Application of CFMR to a real dataset’ further below), ${\tilde{Z}}_{k}$ is the output of the clumped GWAS result of *X* using data with an index in $I_{k}^{c}$.

We then use these Υ_*k*_ variants to build *pred*_*k*_ (a predictor of *X*) and use the data with an index in $I_{k}^{c}$ as a training set. The predictor *pred*_*k*_ can be based on any machine learning/statistical method suitable for building IVs, such as the least absolute shrinkage and selection operator (LASSO) [19] or PRS [20]; in case of non-linear relationship between *Z* and *X*, non-parametric methods such as generalized random forest [21] can be applied. For each *k*, we define the CFI of *X* on *I*_*k*_ as:

$$\begin{array}{c} {\hat{X}}_{k}=pred_{k}\left({\left(Z_{i,1,k},\ldots ,Z_{i,{ϒ}_{k},k}\right)}_{i\in I_{k}}\right) \end{array}$$ (3)

Where *Z*_*i*,*l*, *k*_ is the variant *Z*_*l*,*k*_ of individual *i*. A CFI on *I*_*k*_ is the prediction of *X* on *I*_*k*_ using a predictor of *X* trained using data with an index in $I_{k}^{c}$. Thus, ${\hat{X}}_{k}$ is a vector of length $\frac{N}{K}$. For *i* ∈ *I*_*k*_, we denote the predicted exposure of individual *i* using *pred*_*k*_ as ${\hat{X}}_{k,i}$. Finally, the *K*-fold CFI, $\overset{⌄}{X}$, is a vector of length *N*, where each of its component is defined as:

$$\begin{array}{c} {\overset{⌄}{X}}_{i}={\hat{X}}_{k,i}\;\text{for}\;i\in I_{k} \end{array}$$ (4)

Simply put, $\overset{⌄}{X}$ is a concatenation of the vectors ${\left({\hat{X}}_{k}\right)}_{k\in ⟦1:K⟧}$.

### 2.2 CFMR

**Fig 1:**
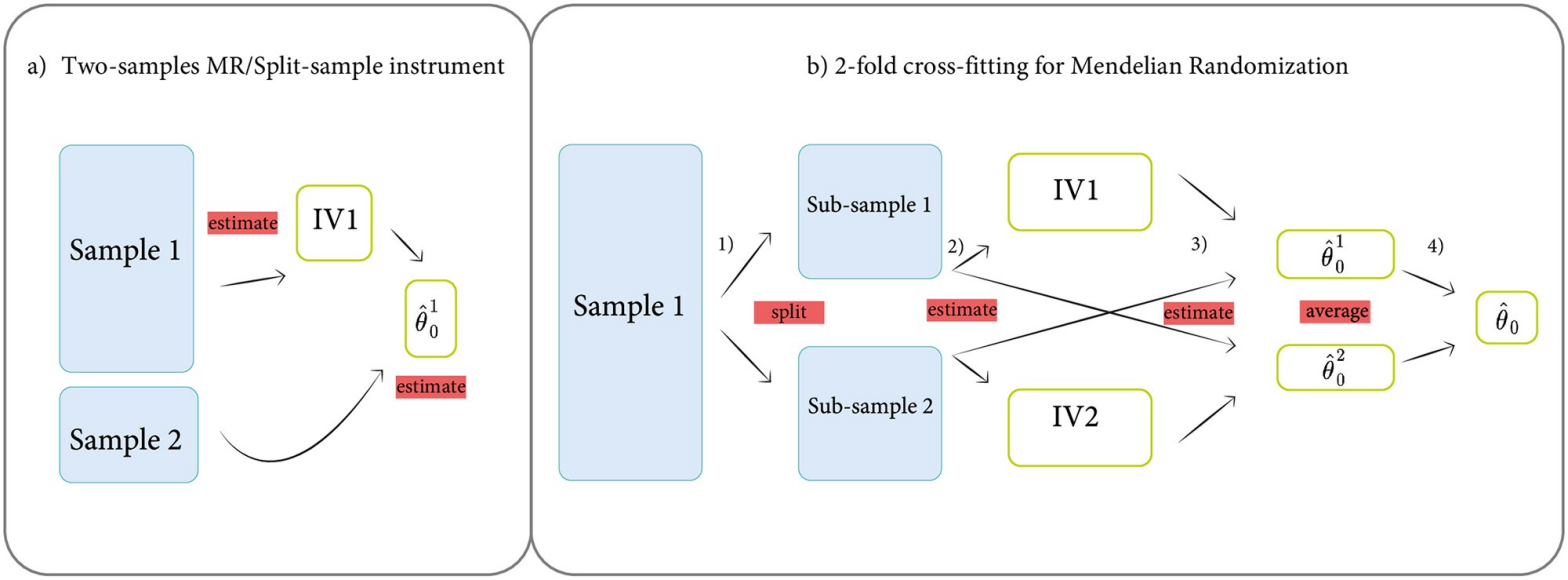
Schematic overview of two-sample MR and two-fold CFMR. Panel (a) shows the two-sample MR setup in which the first sample is used to build the instrument and the second sample is used to estimate the causal effect. Panel (b) shows the two-fold CFMR setup. Step 1 in panel (b) describes the random splitting of the dataset into two sub-samples. In step 2, two separate GWASs are performed: the first using sub-sample 1 and the exposure and the second using sub-sample 2 and the exposure. The predictors of the exposure are subsequently built based on sub-sample 1 (IV1) and sub-sample 2 (IV2). Step 3 refers to the 2SLS in which IV1 is applied to sub-sample 2 and IV2 is applied to sub-sample 1 to obtain the estimates of these IVRs. Finally, in step 4, the two 2SLS from step 3 are simply averaged to obtain the final estimate.

The CFMR1 estimate of *β*_0_ is defined as:

$$\begin{array}{c} {\hat{\beta }}_{0}^{CFMR1}=\frac{1}{K}\sum_{k=1}^{K}2SLS\left(X_{I_{k}},Y_{I_{k}},{\hat{X}}_{k}\right) \end{array}$$ (5)

where 2*SLS* is the 2SLS estimator [22], as in:

$$\begin{array}{c} 2SLS\left(X,Y,Z\right)={\left[X^{t}Z{\left(Z^{t}Z\right)}^{-1}Z^{t}X\right]}^{-1}X^{t}Z{\left(Z^{t}Z\right)}^{-1}Z^{t}Y \end{array}$$ (6)

where the exponent *t* is the transpose operator. This estimate corresponds to the final step (step 4) in panel b in Fig 1. CFMR1 consists of performing an IVR on the partition *I*_*k*_ using ${\hat{X}}_{k}$ as instrument. We then average the estimates of these IVRs to obtain the final estimate.

The CFMR2 estimate of *β*_0_ is simply defined as:

$$\begin{array}{cc} {\hat{\beta }}_{0}^{CFMR2} & =2SLS\left(X,Y,\overset{⌄}{X}\right) \end{array}$$ (7)

$$\begin{array}{l} \begin{array}{c} ={\left[X^{t}\overset{⌄}{X}{\left({\overset{⌄}{X}}^{t}\overset{⌄}{X}\right)}^{-1}{\overset{⌄}{X}}^{t}X\right]}^{-1}X^{t}\overset{⌄}{X}{\left({\overset{⌄}{X}}^{t}\overset{⌄}{X}\right)}^{-1}{\overset{⌄}{X}}^{t}Y \end{array} \end{array}$$ (8)

In essence, CFMR2 consists of performing a single IVR on the entire dataset using $\overset{⌄}{X}$ as instrument. In both CFMR1 and CFMR2, ${\hat{\beta }}_{0}$ is asymptotically normally distributed around *β*_0_ (for details, see [12, 13]).

## Finite sample conservative estimation

As pointed out by Nagar [9], the bias of a 2SLS estimate depends on the strength of the instruments as well as the number of instruments used. In particular, Nagar [9] showed that increasing the number of instruments while keeping the overall strength of the instruments constant results in an increased bias in the 2SLS estimates. The bias due to the number of instruments is proportional to the following quantity:

$$\begin{array}{c} E\left[UZ\hat{\Pi }\right]=E\left[E\left[UZ\hat{\Pi }|Z\right]\right]=E\left[Z{\left(Z^{t}Z\right)}^{-1}\cdot Z^{t}E\left[UV^{t}|Z\right]\right]=E\left[Z{\left(Z^{t}Z\right)}^{-1}Z^{t}\cdot \sigma_{U,V}\right]=\frac{ϒ}{N}\sigma_{U,V} \end{array}$$ (9)

where $\sigma_{U,V}=E\left[UV^{t}|Z\right]$ is the covariance of the error terms in the first- and second-stage regression. Similar to the argument set forth by Angrist *et al.* [13], given that we use a CF procedure, $\hat{X}=Z\hat{\Pi }$ is by design independent of *U*, with the error terms being independent as well, which implies that $E[UZ\hat{\Pi }|Z]=0$. In other words, since the endogeneity bias in 2SLS is proportional to the correlation between the estimates of the first- and second-stage regression, we aim at waiving this bias by setting this correlation to zero.

The results of the simulations are provided in Section 3.1 in the S1 Appendix (see also S1 Fig and S1 Table). The results of the simulations detailed in Section 3 of the S1 Appendix show that CFMR is not unbiased in finite samples. The bias is negligible, as the simulations also show that CFMR1 and CFMR2 are biased toward the null (similar to two-sample MR [23]). This bias toward the null makes CFMR1 and CFMR2 conservative approaches. The Jackknife IV [13] is a special case of CFMR2 for *K* = *N*, and is also biased toward the null in finite samples. However, we show that the bias decreases with increasing sample size. More specifically, the CFMR1 and CFMR2 estimates converge to their true value at a rate of $\frac{\sigma }{\sqrt{(}n)}$ regardless of the strength of the instrument (for details on the convergence results, see [12], proposition 2). The convergence speed of CFMR is the same as that of the standard two-sample MR [24], implying that the two approaches have the same asymptotic power.

Further, we performed an extensive assessment of the behavior of CFMR with regard to how it handles bias, type I error, convergence speed, and statistical power. Specifically, we demonstrate that CFMR is conservative even under extreme scenarios in which standard one-sample MR is heavily biased (see Section 3.2 in the S1 Appendix). Lastly, we describe a simple modification of the CFMR procedure to handle heterogeneity when analyzing a dataset containing multiple ethnicities (Section 3.3 in the S1 Appendix). We show that our modification of CFMR can improve the precision of the 2SLS estimates substantially and provide smaller confidence intervals compared to the standard CFMR.

## 3 Results

### 3.1 Assessment of the statistical properties of CFMR

We assessed the behavior of CFMR in terms of the type I error, bias, convergence speed, and statistical power when LASSO is used to build the exposure predictors *pred*_1_ and *pred*_2_. We refer to CFMR as the estimator CFMR2. Note that we chose CFMR2 over CFMR1 because, as is the case with DML1 and DML2 [11], CFMR2 exhibits a better finite sample size performance than CFMR1. Similar to the simulation setup in Deng *et al.* [24], we consider a set of 300 independent variants (*V*_1_, …., *V*_300_) for each simulation, where each variant has a minor allele frequency of 0.3 and only the first five variants are associated with the exposure. The exposure is generated as $X=\sum_{l=1}^{5}\pi_{l}V_{l}+v$ and the outcome as *Y* = *β*_0_*X* + *u*, where *v* and *u* are two correlated error terms generated from a bivariate normal distribution.

$$\begin{array}{c} \left(\begin{array}{c} U\\ V \end{array})∼N[\begin{array}{cc} (\begin{array}{c} 0\\ 0 \end{array})\;\;, & \left(\begin{array}{cc} 5 & 0.8\\ 0.8 & 5 \end{array}\right) \end{array}\right] \end{array}$$

The variants have the same effect (i.e., *π*_1_ = … = *π*_5_ = *π*), where *π* is selected to ensure that the variants explain *h*^2^ = 20% of the variation in the exposure. Note that our simulation setup differs in two important ways from the one in Deng *et al.* [24]. Firstly, the five variants associated with the exposure are assumed to be known in the simulations by Deng *et al.* [24] (i.e., the authors assume to know which SNP is a valid instrument and the effect it has on the exposure). Whereas Deng *et al.* only use the five truly-associated variants as instruments, we consider a more stringent setup where we purposefully dilute the effects of the five truly-associated variants by adding 295 non-associated variants. Secondly, the amount of confounding is larger in our case. This simulation setup makes it more challenging to construct accurate predictors of the exposure compared to the one by Deng *et al.* [24], particularly when the sample size is small and the IV is weak.

**Fig 2:**
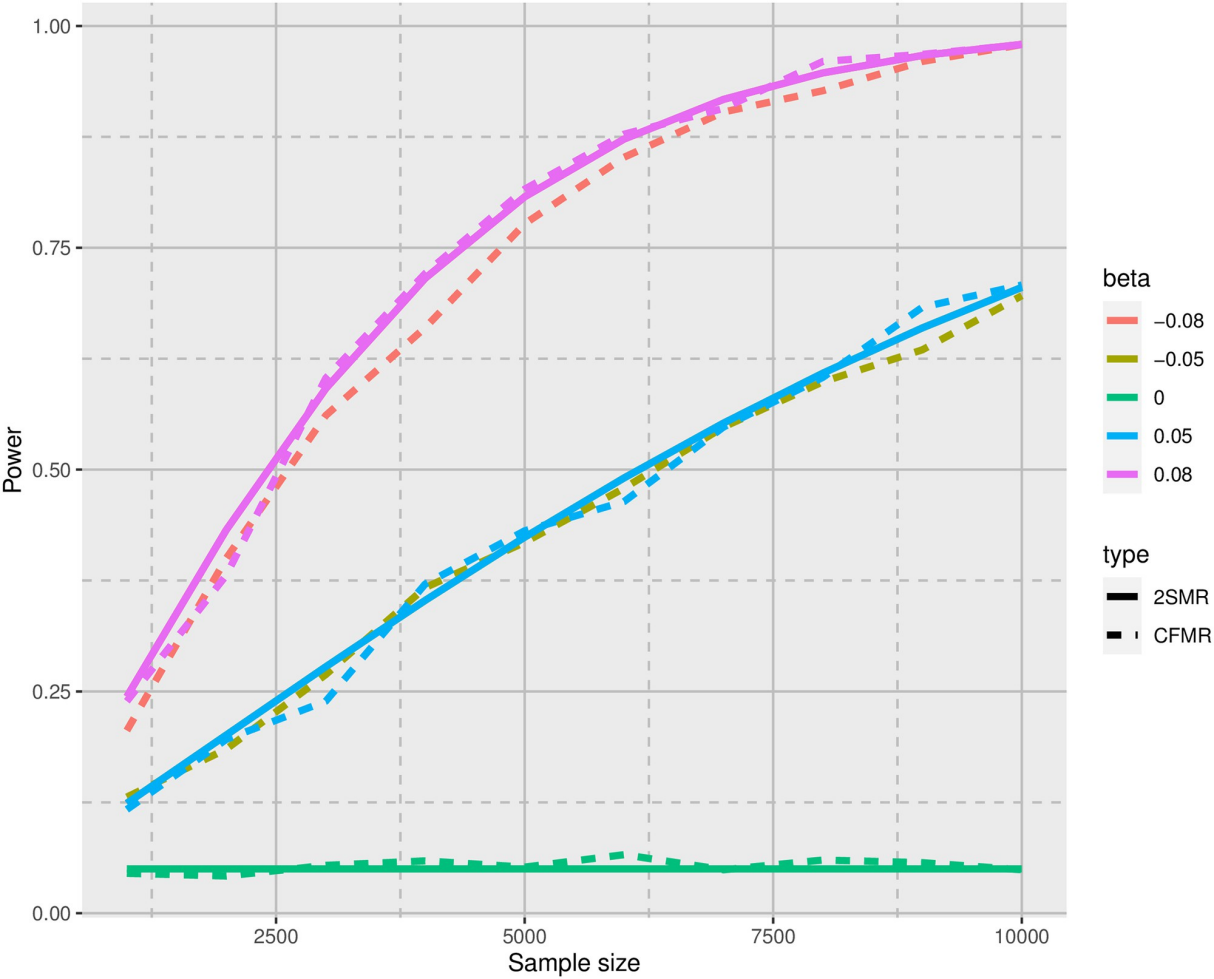
Power curves for CFMR versus two-sample MR (2SMR) using the simulation setup described in the Simulations section in the main text (with *h*^2^ = 20%). The dashed lines represent power curves for CFMR, while the solid lines represent the theoretical power for 2SMR [24]. Note that the solid pink line covers the solid red line perfectly (top part of the graph). These lines fully overlap as a result of symmetry. Given that the solid lines are generated from the theoretical power formula of two-sample MR (see Deng *et al.* [24]), the red and pink curves correspond to the same effect size (in terms of magnitude) but have opposite signs. The same is the case for the solid blue and gold lines in the middle part of the graph.

We applied CMFR to each simulated dataset using a LASSO-based IV after applying ten random splits. We considered various sample sizes (N = 1, 000 to 10, 000) and different *β*_0_ values (-0.08, -0.05, 0, 0.05, and 0.08), similar to the simulations by Deng *et al.* [24]. For each combination of sample and effect size, we simulated 1000 datasets. Fig 2 summarizes the results of our simulations; we also provide a numerical summary of these simulations in S1 and S2 Tables. Further we assessed the type I error, bias, and power of CFMR for different estimates of the variance explained by the exposure (*h*^2^ = 0%, 0.001%, 0.01%, 0.1%, 1%, 5%, and 10%) and different sample sizes (N = 1, 000, 5, 000, 10, 000, 50, 000, 100, 000, and 500, 000). For large sample sizes (e.g., N = 100, 000 or N = 500, 000), we were unable to perform as many simulations as with the smaller samples. We provide a numerical summary of these simulations in S3 and S4 Tables. Simulations were performed on a computer cluster with 32 CPUs and 128 GB RAM.

### 3.2 Comparison with competing approaches

In this section, we perform a number of simulations to show that current MR approaches that can account for weak instrument bias, such as MR RAPS [25] and the method by Barry *et al.* [26], are heavily biased when using completely ‘overlapping’ datasets (which, in the current context, means using a single dataset). CFMR, on the other hand, remains conservative. The simulations were performed as follows. For each simulation, we consider a set of 300 independent variants (*V*_1_, …., *V*_300_), where each variant has a minor allele frequency of 0.3 and only the first five variants are associated with the exposure. As mentioned in Section 3.1 above, these criteria are similar to the simulation setup in Deng *et al.* [24]. The exposure is generated as $X=\sum_{l=1}^{5}\pi V_{l}+v$ and the outcome as *Y* = *β*_0_*X* + *u*, where *v* and *u* are two correlated error terms generated from a bivariate normal distribution.

$$\begin{array}{c} \left(\begin{array}{c} U\\ V \end{array})∼N[\begin{array}{cc} (\begin{array}{c} 0\\ 0 \end{array})\;\;, & \left(\begin{array}{cc} 5 & 4.9\\ 4.9 & 5 \end{array}\right) \end{array}\right] \end{array}$$

**Fig 3:**
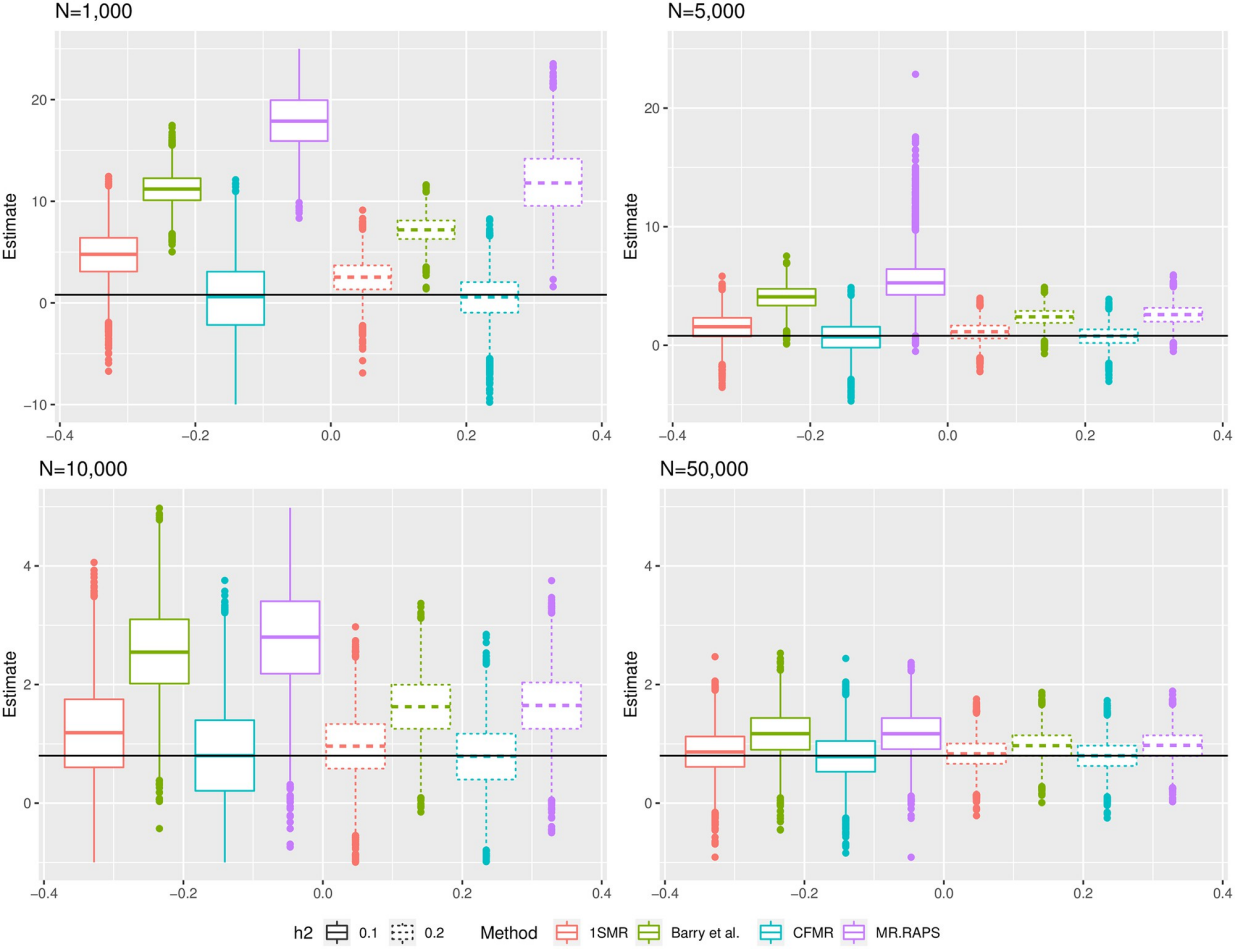
Summary of the results of the simulations to assess bias due to complete sample overlap and weak instruments across different MR methods. For details, see Section 3.2 in the main text. Each panel displays the box plots of the estimated effect according to method used (1SMR, Barry *et al.* [26], CFMR, and MR RAPS) and sample size (1000, 5, 000, 10, 000, and 50, 000). The y-axis corresponds to the estimated effect. The solid horizontal black line corresponds to the true value of the effect to be estimated. The different types of box plots correspond to the variance *X* explained by the genetic marker used as instruments (10% and 20%). The red box plots correspond to the estimates based on one-sample MR, the green box plots the estimates based on the Barry *et al.* [26] method, the purple box plots the estimate using MR RAPS, and finally, the blue box plots the estimates using CFMR.

We consider the following two scenarios: 1) the variants explain 10% of the variance of *X* (*h*^2^ = 10%), and 2) the variants explain 20% of the variance of *X* (*h*^2^ = 20%). On each simulated dataset, we apply CFMR using a LASSO-based IV and ten random splits. The same was applied to MR RAPS [25] and the Barry *et al.* method [26]. We also build the predictor *pred*_*all*_ of *X* using LASSO and the entire dataset. We then use the prediction *pred*_*all*_ on the entire data as instrument. We refer to ‘one-sample MR estimates’ when estimating the effect of *X* on *Y* using the prediction of *pred*_*all*_. We consider various sample sizes, ranging from 1, 000 to 50, 000, and set *β*_0_ = 0.08. For each combination of sample and effect size, we simulate 1000 datasets. The results of these simulations are summarized in Fig 3 and S7 Table.

The results of our simulations show that CFMR remains conservative when using a single dataset to perform an MR analysis (see Fig 3), whereas the other approaches show substantial bias. Notably, the bias from MR RAPS and the Barry *et al.* method seems to depend on the strength of the instrument, whereas CFMR does not display any bias due to instrument strength. Furthermore, it is clear from Fig 2 that CFMR and two-sample MR have very similar power. CFMR also shows excellent control of the type I error for the different nominal levels tested (see S2 Table and S4 Fig).

## 4 Application of CFMR to a real dataset

We applied CFMR2 to a dataset of mother-newborn dyads from the Norwegian Mother, Father, and Child Cohort Study (MoBa) [27]. Our objective was to re-examine the well-established effect of maternal pre-pregnancy BMI on newborn’s birth weight [28]. We chose CFMR2 over CFMR1 because, as is the case with DML1 and DML2 (see [11]), CFMR2 exhibits a better finite sample size performance than CFMR1. After applying the quality control criteria outlined in Section 4 in the S1 Appendix, 26, 896 complete mother-newborn dyads with genotype and phenotype data remained for the current analyses. As additional criteria, we assumed random mating between parents and independence between mothers (i.e., no sibships) [29]. The maternal genotype was used to build the instrument for pre-pregnancy BMI. CFMR was run on the 26, 896 mother-newborn dyads using 10 random splits; i.e., 10 separate GWASs of pre-pregnancy BMI were performed, with each GWAS encompassing 24, 210 randomly selected mothers (approx. 90% of the original 26, 896 mothers). As our sample is relatively modest in size, we only used the first three principal components (PCs) to adjust for population stratification in each GWAS.

The Manhattan plots of the 10 GWASs are provided in Figs S9-S18 in the S1 Appendix. The results across the GWASs are similar and show a systematic replication of the top hits previously identified by two large GWAMAs of BMI [30, 31]. These include SNPs in the genes *FTO*, *TMEM18*, and *MC4R*. Furthermore, we clumped the results of each GWAS using PLINK version 1.9 [32] and used *r*^2^ = 0.1 within a 500-kb window as criterion. We then selected SNPs with a P-value below 10^−6^ and used LASSO to build a predictor of maternal pre-pregnancy BMI. The most fitting λ parameter for the LASSO was determined by cross-validation using the *cv.glmnet* function from the ***R*** package *glmnet* [33]. The next step was to build the CFI by predicting each mother’s pre-pregnancy BMI using a predictor trained on a dataset that does not contain the data to be predicted. To assess the effect on the CFMR estimate of using different thresholds for including SNPs in our CFI, we repeated the above procedure using different P-value thresholds ranging from 10^−3^ to 10^−8^ to build a CFI for each of these thresholds.

In addition, to contrast CFMR with one-sample MR, we also performed a GWAS of pre-pregnancy BMI using the entire dataset of 26, 896 mother-newborn dyads in MoBa. Again, we clumped the results of the GWAS and selected SNPs with a P-value below 10^−6^ to build a predictor of maternal pre-pregnancy BMI by applying LASSO to the entire dataset. We then used the prediction of the predictor of maternal pre-pregnancy BMI as instrument. We refer to the estimation of the effect of maternal pre-pregnancy BMI on birth weight as ‘one-sample MR estimation’. Similar to the analyses above for the truncated dataset, we used different P-value thresholds ranging from 10^−3^ to 10^−8^ and estimated the effect of maternal pre-pregnancy BMI for each of these thresholds.

Finally, we also performed a two-sample MR estimation of the effect of maternal BMI on birth weight to compare with the CFMR results. This was done using the same SNPs and *β* values as in Tyrrell *et al.* [28] to generate a PRS for maternal BMI. We then performed a 2SLS to evaluate the effect of maternal pre-pregnancy BMI on birth weight using the PRS for pre-pregnancy maternal BMI as instrument. As these SNPs and regression coefficients were determined using the results from a published GWAMA on BMI [34] (*n* = 123, 865) that did not include the current dataset from MoBA, any bias due to sample overlap is minimized in our two-sample MR [5].

### 4.1 Application results

Except for the CFI constructed using SNPs with a P-value below 10^−8^, CFIs of maternal pre-pregnancy BMI explained about 1% of the variance in pre-pregnancy BMI (Table 1). When testing the CFIs for association with potential confounders, such as maternal age and pre-pregnancy maternal smoking [28], we found no evidence of an association between these variables and the CFIs constructed using SNPs with a P-value below 10^−6^. By contrast, for CFIs constructed using SNPs with a P-value threshold larger than 10^−6^, we observed moderate to strong associations with these variables. The results are summarized in S6 Table.

**Table 1:** CFMR estimates of maternal pre-pregnancy BMI on newborn’s birth weight per 1 SD increase in maternal pre-pregnancy BMI.

−*log*_10_ SNP P-value	1SMR estimate	1SMR Std. error	CFI variance explained (%)	CFMR estimate	CFMR Std. error	P-value	95% CI	SNPs per split
-3	84.4	4.4	1.112	101.6	25.0	0.00005	52.6–150.6	1798
-4	88.6	5.8	1.102	113.8	26.3	0.00002	62.2–165.5	624
-5	88.1	8.4	1.101	94.3	26.6	0.00038	42.3–146.4	198
-6	94.5	12.3	1.112	82.4	24.9	0.00093	33.6–131.2	52
-7	85.6	16.3	0.951	73.4	27.0	0.00657	20.5–126.2	22
-8	108.1	19.2	0.044	87.0	38.4	0.02351	11.7–162.3	6

‘−*log*_10_ SNP P-value’ corresponds to the cutoff used to build the CFI.

‘1SMR estimate’ corresponds to the estimation using one-sample MR.

‘1SMR Std. error’ corresponds to the standard error of the estimation based on one-sample MR.

‘Variance explained’ corresponds to the pre-pregnancy variance explained by the CFI.

‘SNPs per fold’ corresponds to the average number of SNPs with a P-value below a given threshold after clumping the output of each GWAS of maternal pre-pregnancy BMI.

‘Selected SNPs per fold’ corresponds to the average number of SNPs selected by LASSO to build the instrument in each fold.

Abbreviations: 1SMR, one-sample MR; CFI, Cross-Fitted Instrument; Std. error, standard error; CFMR, Cross-Fitting for Mendelian Randomization CI, confidence interval;

For each CFI, we performed 2SLS to estimate the causal effect of maternal pre-pregnancy BMI on newborn’s birth weight. To follow the approach of Tyrrell *et al.* [28], we also adjusted for maternal age and fetal sex in each of these 2SLS. The CFMR2 estimates remained similar across the different CFIs (Table 1 and S5 and S21 Figs). Table 1 summarizes the CFMR estimates generated using the CFI based on SNPs with a P-value below 10^−7^. We used this CFI because it explained a relatively large fraction of maternal pre-pregnancy BMI (0.91%) and was not associated with any of the potential confounders.

The results of our CFMR analyses indicated that a genetically-predicted increase of 1 SD of maternal pre-pregnancy BMI ($4.2\frac{kg}{m^{2}}$) was associated with an increase in newborn’s birth weight of 73.35 g (95% CI: 20.46 − 126.24, *P* = 6.56 × 10^−6^), which corresponds to an increase in newborn’s birth weight of 17.42 g (95% CI: 4.86 − 29.98, *P* = 6.56 × 10^−6^) per unit increase in genetically-predicted maternal pre-pregnancy BMI. These CFMR estimates are similar to the observational associations in our dataset; that is, of an increase of 81.90 g (95% CI: 75.93 − 87.88, *P* < 10^−16^) in newborn’s birth weight per 1 SD higher maternal pre-pregnancy BMI, or an increase of 19.44 g (95% CI: 18.03 − 20.87, *P* < 10^−16^) per unit increase of genetically-predicted maternal pre-pregnancy BMI. Lastly, our two-sample MR results indicated that a genetically-predicted increase of 1 SD of maternal pre-pregnancy BMI ($4.2\frac{kg}{m^{2}}$) was associated with an increase in newborn’s birth weight of 71.74 g (95% CI: 39.08 − 104.40, *P* = 1.67 × 10^−5^), which corresponds to an increase in newborn’s birth weight of 11.12 g (95% CI: 6.06 − 16.18, *P* = 1.67 × 10^−5^) per unit increase of genetically-predicted maternal pre-pregnancy BMI.

## 5 Discussion

This study presents a new type of IV, termed CFI, that is readily adaptable to an MR setting. The main advantage of CFMR over regular two-sample MR is its ability to perform a two-sample MR using a single sample, allowing its application to considerably smaller sample sizes than is currently feasible with conventional two-sample MR methods. Furthermore, CFMR ensures that the population assumptions of MR are satisfied. Additional advantages of CFMR include affording the same power as a two-sample MR and allowing estimates from multiple CFMRs to be meta-analyzed while taking into account heterogeneity between the different estimates. As CFMR is modular, it lends itself easily to parallel-computing and can therefore be used in conjunction with many statistical methods to build multiple variant scores for downstream analyses, such as PRS [35] or LASSO-based instruments [19, 36–38].

**Fig 4:**
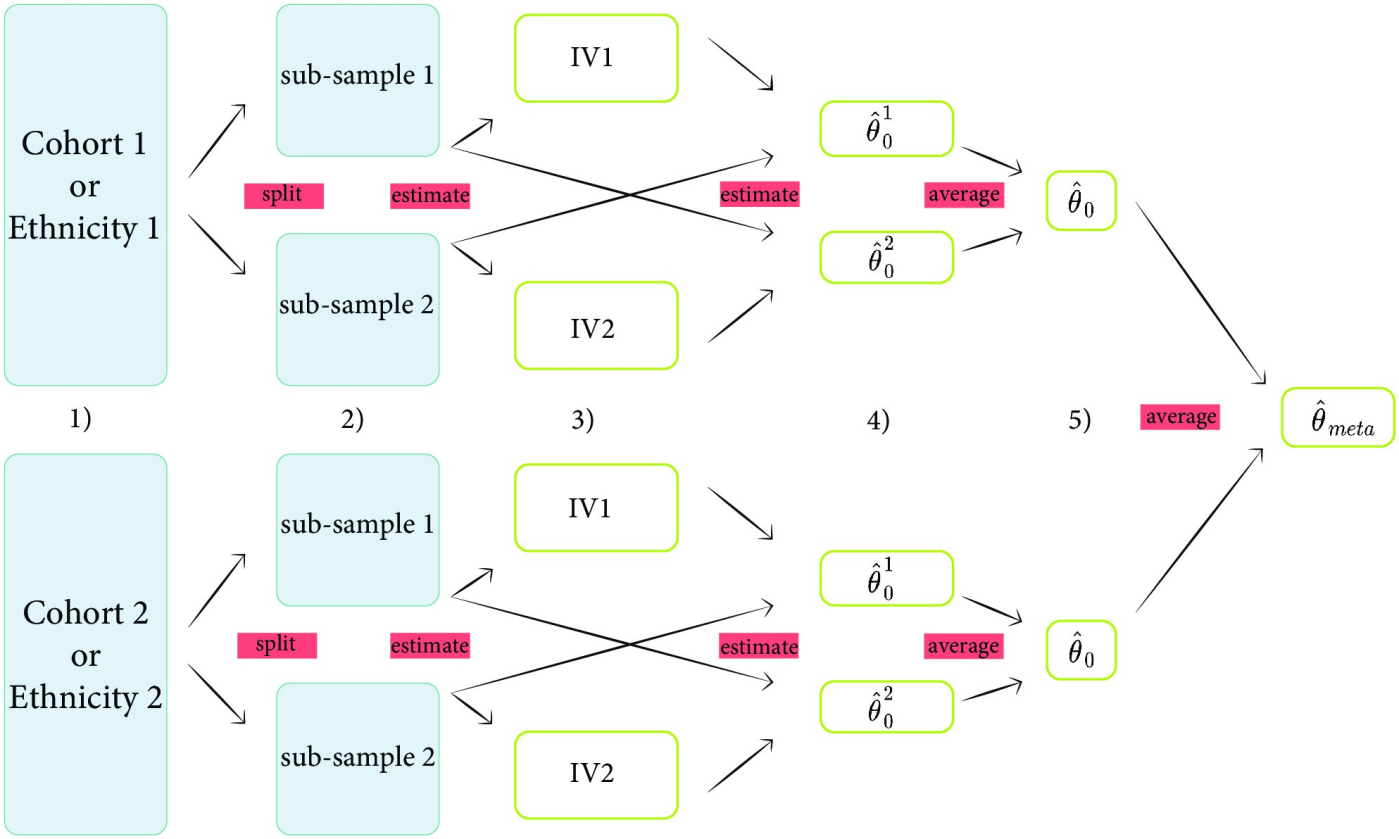
Schematic overview of the application of CFMR to a dataset comprising two ethniticies. In step 1, the two ethnicities are first separated into two distinct datasets, where each dataset contains individuals of the same ethnicity. In step 2, the dataset is split at random for each ethnicity. In step 3, two separate GWASs are performed: the first using sub-sample 1 and the exposure and the second using sub-sample 2 and the exposure. The predictors of the exposure are subsequently built based on sub-sample 1 (IV1) and sub-sample 2 (IV2). Step 4 refers to the 2SLS using IV1 on sub-sample 2 and IV2 on sub-sample 1, and, for each dataset, the two 2SLS from step 3 are averaged. Finally, in step 5 the two estimates are meta-analyzed to obtain the final estimate.

Compared to two-sample MR, in which one sample is used to build the instrument and the other sample to test for association, CFMR allows a conservative estimation of causal effects using two samples from the same source population. If two separate samples are available for analysis, CFMR can be applied to each sample separately followed by a meta-analysis of the results. As meta-analyzing the results increases the active sample size of the study compared to a two-sample MR, CFMR can potentially enhance statistical power in MR analyses. Similarly, CFMR can easily handle multiple ethnicities in the same sample by building a CFI for each ethnicity and aggregating these into a cross-ethnic CFI (see Fig 4 and Section 2 in the S1 Appendix and S23 Fig and S8 Table). This type of cross-ethnic MR enables accounting for the heterogeneous genetic architecture of the outcome across different study sub-populations. An example of such an outcome is color blindness, which is substantially more heritable in males than females. In particular, our results showed that cross-population CFMR is conservative even when applied to a heterogeneous population. Moreover, it can be up to 37-fold more precise than methods that disregard population heterogeneity.

Chernozhukov *et al.* [11] reported that the number of splits has negligible impact on the asymptotic convergence speed of the DLM methods. Interestingly, our data also showed that the number of splits had no appreciable effect on the convergence speed of the exposure predictors in CFMR. However, as the power of 2SLS depends heavily on the prediction accuracy of the IV [24], it is critical to obtain as good of a genetic predictor of the exposure as possible. For instance, two to five splits may be sufficient if a large sample size (≥ 100, 000) is available for analysis and the exposure is highly heritable (e.g., if a few SNPs have large individual effects on the exposure), but for more complex traits and smaller sample sizes, it may be better to increase the number of splits to improve the predictive performance of the exposure predictors [11]. As a rule of thumb, we recommend using CFMR with 10 splits. In case the exposure is particularly difficult to predict, or the sample size is limited (≤ 5000), or both, using 20–30 splits may provide some improvement. In addition, we recommend using CFMR2 in most practical settings, as also recommended for DML1 versus DML2 by Chernozhukov *et al.* [11]. The rationale for this is that CFMR1 is asymptotically equivalent to CFMR2, but as CFMR1 is an average of estimates based on small datasets, which can be noisy, CFMR1 tends to be less powerful than CFMR2 for small sample sizes. In our presentation of CFMR, we suggest clumping the GWAS results prior to building the IV. However, the application of other steps, such as co-localization [39, 40] or whole-genome (LASSO) regression [41], may also be worth pursuing in this context.

Our simulations indicate that CFMR remains unbiased as long as the sample size is sufficiently large (≥ 100, 000). When the variance explained by the instruments is small, e.g., when *h*^2^ ≤ 1%, CFMR is biased toward the null, which makes it a conservative approach for causal estimation. Therefore, non-significant results based on relatively small sample sizes or very weak instruments are likely to be false-negatives and should therefore not be disregarded from future investigations. Another attractive feature of CFMR is its good control of type I error, even when no instrument is associated with the exposure (i.e., *h*^2^ = 0). This tight error control is partially due to the standard errors of CFMR being too large for small sample sizes (≤ 10, 000), which is not unexpected considering that the standard error for 2SLS estimates is based on normal approximation, which may not be valid for small sample sizes [22]. Obtaining narrower confidence intervals for weak CFIs may increase the power of CFMR for analyzing complex traits and exposures that have low heritability *a priori*.

Future research should focus on deriving the CFMR distribution for weak instruments using robust variance estimates. Aside from analytical challenges inherent to limited sample size, our simulations showed bias toward the null for weak instruments (*h*^2^ ≤ 1%), which is as expected given that we do not assume to know the causal variant *a priori*. For instance, when the total variance explained by the SNPs is 0.1%, each SNP explains only around 0.02% of the exposure heritability. Therefore, it becomes challenging for LASSO [36] to select causal variants among the 300 variants used in our simulations. As with many other MR-based methods, CFMR is not immune to pleiotropy. In future developments of CFMR, we plan to design a one-sample MR method that accounts for endogeneity bias and is robust against pleiotropy (using recent insights from, for example, [18, 42]).

In our application of CFMR to real data, we estimated the effect of maternal pre-pregnancy BMI on newborn’s birth weight in the Norwegian MoBa study. Predictors of maternal pre-pregnancy BMI explained, on average, 11.4% of the variance in maternal pre-pregnancy BMI in the training sets (Supplementary S10–S20 Figs). In comparison, our CFI based on SNPs with a P-value below 10^−7^ explained only 0.915% (P-value ≤ 10^−16^) of the variance. The difference in variance explained by the CFIs in the training versus test set illustrates how CFMR can circumvent the problem of overfitted instruments that can lead to endogeneity bias [5] (see also Supplementary S9–S20 Figs). The variance explained by our CFIs is smaller (between 0.95% and 1.11%) than the variance explained by the IVs used by Tyrrell *et al.* (1.8%) [28]. Interestingly, our estimates of maternal pre-pregnancy BMI on newborn’s birth weight were similar to those reported by Tyrrell *et al.* [28] and to the ones we obtained using a two-sample MR in the MoBa sample. Notably, in the Tyrrell *et al.* study, 1 SD increase in maternal pre-pregnancy BMI ($4\frac{kg}{m^{2}}$ in Tyrrell *et al.* and $4.2\frac{kg}{m^{2}}$ in our analysis) corresponded to an increase of 55 g (95% CI: 17 − 93) in newborn’s birth weight (compared to 73.35 g (95% CI: 20.46 − 126.25) in our analysis).

In the Tyrrell *et al.* study, the observational association corresponded to an increase of 62 g (95% CI: 56 − 70) in newborn’s birth weight per 1 SD of higher maternal pre-pregnancy BMI. Furthermore, the confidence intervals in the Tyrrell *et al.* study were narrower than ours. This is because the IVs used by Tyrrell *et al.* explained 1.8% of the pre-pregnancy maternal BMI variance compared to 0.95% in our case. Nonetheless, the IVs in Tyrrell *et al.* were constructed using the results of a previously published GWAMA of BMI [34] that is approximately five-fold larger than our dataset. Therefore, the overall two-sample MR of Tyrrell *et al.* required a sample size approximately six times larger than ours. In their analysis, Tyrrell *et al.* used 123, 865 genotyped individuals to identify the instruments and 25, 265 genotyped individuals to estimate the effect of pre-pregnancy maternal BMI on birth weight. In contrast, our CFMR estimate was based on 26, 896 genotyped individuals. This illustrates how CFMR can provide accurate causal estimates using substantially smaller datasets.

As recently pointed out by Zhang *et al.* [6], variations across populations may lead to biased estimates in an MR analysis. It is interesting to observe that our CFMR estimates were similar to those of the one-sample MR. The fact that the CFMR estimates were similar to the observational associations points to minimal confounding in the analyses. It is, therefore, not unexpected that the one-sample MR estimates would be similar to the CFMR estimates (see Table 1 and S21 Fig). However, the standard errors of one-sample MR estimates were smaller than those of the CFMR estimates. These small standard errors are likely due to the inability of one-sample MR to handle overfitting, which results in an overly confident estimation (S21 Fig) and biased inference. We illustrate this bias using simulations, and the results indicated that one-sample MR RAPS [25] and the Barry *et al.* method [26] can be heavily biased even when the instrument is strong (*h*^2^ > 20%, see Fig 3). CFMR, on the other hand, remains conservative in the presence of weak instruments and strong confounding.

To conclude, we show that CFMR is a valuable new approach for MR analysis, particularly for small sample sizes and understudied exposures. It is especially useful for investigating exposures and outcomes that might be difficult or expensive to measure, or when dealing with populations consisting of multiple ethnicities. Moreover, CFMR has the potential to enhance statistical power of two-sample MR in consortia-led meta-analyses, where each cohort can apply CFMR to its study population and the results from each cohort subsequently meta-analyzed in the final step of the analysis. Our results showed that CFMR performed particularly well when the sample is sufficiently large (≥ 100, 000) and even when the instruments are weak (*h*^2^ ≤ 1%). These advantageous features make CFMR an attractive tool to reassess the causal effect of poorly heritable traits, especially those with genotype data accessible through various public repositories, such as the Database of Genotypes and Phenotypes (dbGaP, https://www.ncbi.nlm.nih.gov/gap/) or the UK Biobank (https://www.ukbiobank.ac.uk/).

## Software

A typical CFMR run is provided as an R script at https://github.com/william-denault/CFMR. The scripts used for the current simulations and application to maternal pre-pregnancy BMI have also been deposited there.

## Supporting information

S1 Appendix Details of the additional simulations and description of the cross-population CFMR. (PDF)

S1 Fig Power of CFMR vs. 2SMR. Power curves for CFMR versus two-sample MR (2SMR) using the simulation setup described in the Simulations section in the main text (with *h*^2^ = 20%). The dashed lines represent power curves for CFMR and the solid lines represent the theoretical power for 2SMR [24]. Note that the solid pink line covers the solid red line perfectly. These lines fully overlap as a result of symmetry and both lines correspond to the same effect size but have opposite signs. The same is the case for the solid blue and gold lines.
(TIF)

S2 Fig Mean estimates of *β*_0_ by CFMR for different values of *h*2 and sample size. Mean estimate of beta by CFMR against the true beta for different values of beta, *h*^2^ and N.
(TIF)

S3 Fig CFMR standard errors for different values of *h*2 and sample size. Empirical standard deviation of CFMR against the mean of the estimated standard deviations of CFMR for different values of beta, *h*^2^ and N.
(TIF)

S4 FigMean of the estimated standard deviations of CFMR.Mean of the estimated standard deviations of CFMR for different values of beta, *h*^2^ and N. Each configuration was simulated 1000 times. The solid line is the function $f\left(x\right)=\frac{\sigma }{sqrt(x)}$, where *σ*^2^ is the variance of Υ in the simulations described in Section 3.1.(TIF)

S5 FigCFMR convergence speed for *h*2 = 20%.The black dots correspond to the empirical standard deviation of CFMR for various values of *β*_0_. The red dots correspond to the empirical standard deviation of CFMR for various values of *β*_0_ (*h*^2^ = 20%). The black line corresponds to the function $f\left(x\right)=\frac{\sigma }{sqrt(x)}$, where *σ*^2^ is the variance of the simulations described in Section 3.1.(TIF)

S6 Fig CFMR standard error for *h*2 = 20% and different sample sizes. Empirical standard deviation of CFMR against its theoretical standard deviation for different values of *β*_0_ and N, with *h*^2^ = 20%.
(TIF)

S7 Fig Density of CFMR estimates for *h*2 = 0% and different sample sizes. Density of the estimation of CFMR when no instrument is causally related to the exposure, based on different sample sizes.
(TIF)

S8 Fig Density of CFMR estimates for different values *h*2% and different sample sizes. Density of the estimation of CFMR when *β*_0_ = 0 for different sample sizes. The variance explained by the instrument (*h*^2^) is displayed on top of each plot.
(TIF)

S9 Fig Boxplot of predicted pre-pregnancy BMI performance; P-value threshold 10^−3^. Predicted pre-pregnancy BMI performance on test and training sets. Left panel: the boxplot of the difference between the predicted pre-pregnancy BMI and the observed pre-pregnancy BMI on each training set, using a P-value threshold of 10^−3^. Right panel: the boxplot of the difference between the predicted pre-pregnancy BMI and the observed pre-pregnancy BMI on each test set, using a P-value threshold of 10^−3^.
(TIF)

S10 Fig Boxplot of predicted pre-pregnancy BMI performance; P-value threshold 10^−4^. Predicted pre-pregnancy BMI performance on test and training sets. Left panel: the boxplot of the difference between the predicted pre-pregnancy BMI and the observed pre-pregnancy BMI on each training set, using a P-value threshold of 10^−4^. Right panel: the boxplot of the difference between the predicted pre-pregnancy BMI and the observed pre-pregnancy BMI on each test set, using a P-value threshold of 10^−4^.
(TIF)

S11 Fig Boxplot of predicted pre-pregnancy BMI performance; P-value threshold 10^−5^. Predicted pre-pregnancy BMI performance on test and training sets. Left panel: the boxplot of the difference between the predicted pre-pregnancy BMI and the observed pre-pregnancy BMI on each training set, using a P-value threshold of 10^−5^. Right panel: the boxplot of the difference between the predicted pre-pregnancy BMI and the observed pre-pregnancy BMI on each test set, using a P-value threshold of 10^−5^.
(TIF)

S12 Fig Boxplot of predicted pre-pregnancy BMI performance; P-value threshold 10^−6^. Predicted pre-pregnancy BMI performance on test and training sets. Left panel: the boxplot of the difference between the predicted pre-pregnancy BMI and the observed pre-pregnancy BMI on each training set, using a P-value threshold of 10^−6^. Right panel: the boxplot of the difference between the predicted pre-pregnancy BMI and the observed pre-pregnancy BMI on each test set, using a P-value threshold of 10^−6^.
(TIF)

S13 Fig Boxplot of predicted pre-pregnancy BMI performance; P-value threshold 10^−7^. Predicted pre-pregnancy BMI performance on test and training sets. Left panel: the boxplot of the difference between the predicted pre-pregnancy BMI and the observed pre-pregnancy BMI on each training set, using a P-value threshold of 10^−7^. Right panel: the boxplot of the difference between the predicted pre-pregnancy BMI and the observed pre-pregnancy BMI on each test set, using a P-value threshold of 10^−7^.
(TIF)

S14 Fig Boxplot of predicted pre-pregnancy BMI performance; P-value threshold 10^−8^. Predicted pre-pregnancy BMI performance on test and training sets. Left panel: the boxplot of the difference between the predicted pre-pregnancy BMI and the observed pre-pregnancy BMI on each training set, using a P-value threshold of 10^−8^. Right panel: the boxplot of the difference between the predicted pre-pregnancy BMI and the observed pre-pregnancy BMI on each test set, using a P-value threshold of 10^−8^.
(TIF)

S15 Fig Predicted pre-pregnancy BMI performance on test and training sets; P-value threshold 10^−3^. Predicted pre-pregnancy BMI performance on test and training sets. Left panel: the bivariate plot of the predicted pre-pregnancy BMI on training sets against true values using a P-value threshold of 10^−3^. Right panel: the bivariate plot of the predicted pre-pregnancy BMI on test sets against the true values using a P-value threshold of 10^−3^.
(TIF)

S16 Fig Predicted pre-pregnancy BMI performance on test and training sets; P-value threshold 10^−4^. Predicted pre-pregnancy BMI performance on test and training sets. Left panel: the bivariate plot of the predicted pre-pregnancy BMI on training sets against true values using a P-value threshold of 10^−4^. Right panel: the bivariate plot of the predicted pre-pregnancy BMI on test sets against the true values using a P-value threshold of 10^−4^.
(TIF)

S17 Fig Predicted pre-pregnancy BMI performance on test and training sets; P-value threshold 10^−5^. Predicted pre-pregnancy BMI performance on test and training sets. Left panel: the bivariate plot of the predicted pre-pregnancy BMI on training sets against true values using a P-value threshold of 10^−5^. Right panel: the bivariate plot of the predicted pre-pregnancy BMI on test sets against the true values using a P-value threshold of 10^−5^.
(TIF)

S18 Fig Predicted pre-pregnancy BMI performance on test and training sets; P-value threshold 10^−6^. Predicted pre-pregnancy BMI performance on test and training sets. Left panel: the bivariate plot of the predicted pre-pregnancy BMI on training sets against true values using a P-value threshold of 10^−6^. Right panel: the bivariate plot of the predicted pre-pregnancy BMI on test sets against the true values using a P-value threshold of 10^−6^.
(TIF)

S19 Fig Predicted pre-pregnancy BMI performance on test and training sets; P-value threshold 10^−7^. Predicted pre-pregnancy BMI performance on test and training sets. Left panel: the bivariate plot of the predicted pre-pregnancy BMI on training sets against true values using a P-value threshold of 10^−7^. Right panel: the bivariate plot of the predicted pre-pregnancy BMI on test sets against the true values using a P-value threshold of 10^−7^.
(TIF)

S20 Fig Predicted pre-pregnancy BMI performance on test and training sets; P-value threshold 10^−8^. Predicted pre-pregnancy BMI performance on test and training sets. Left panel: the bivariate plot of the predicted pre-pregnancy BMI on training sets against true values using a P-value threshold of 10^−8^. Right panel: the bivariate plot of the predicted pre-pregnancy BMI on test sets against the true values using a P-value threshold of 10^−8^.
(TIF)

S21 Fig CFMR and one-sample MR estimates of pre-pregnancy maternal BMI effect on birth weight. CFMR and one-sample MR (1SMR) estimates of the effect of pre-pregnancy maternal BMI on birth weight, with 95% confidence intervals.
(TIF)

S22 Fig Bias comparison between one-sample MR, MR RAPS, the method by Barry *et al.*, and CFMR. Summary of the simulations performed in Section 3.2. The x-axis corresponds to the number of individuals used in each simulation (1000; 5, 000; 10, 000; and 50, 000), and the y-axis corresponds to the estimated effect. The solid horizontal black line corresponds to the true value of the effect to be estimated. The dashed and solid lines lines correspond to the variance *X* explained by the genetic marker used as instrument (10% and 20%). The different types of lines correspond to the variance *X* explained by the genetic marker used as instruments (10% and 20%). The red lines correspond to the mean estimate using one-sample MR. The green lines correspond to the mean estimate using the Barry et al. method. The pink lines correspond to the mean estimate using MR RAPS and the blue lines correspond to the mean estimate using CFMR.
(TIF)

S23 Fig Cross-population CFMR vs CFMR. Panel a: the blue density represents the density of the cross-population CFMR (labeled as ‘cross pop CFMR’ in the figure) estimates of the parameter of interest in the simulation described in Section 3.3 for n = 1000. The vertical line corresponds to the true value of the parameter of interest, here equal to 1. The red density represents the density of the CFMR estimates of the parameter of interest in the simulation described in Section 3.3 for n = 1000. Panels b, c and d are the same as Panel a, except that n = 5000, 10,000 and 50,000 in each of these, respectively.
(TIF)

S1 Table Power CFMR for *h*^2^ = 20%. Estimated power of CFMR for different values of *β*_0_ and different sample sizes, with *h*^2^ = 20%.
(PDF)

S2 Table Type I error of CFMR, *h*^2^ = 20%. Type I error of CFMR for different sample sizes, with *h*^2^ = 20%.
(PDF)

S3 Table Power CFMR in large sample. Power of CFMR for different sample sizes and values of *h*^2^.
(PDF)

S4 Table Type I error of CFMR in large samples. Type I error of CFMR for different sample sizes and values of *h*^2^.
(PDF)

S5 Table CFMR estimates (raw scale). (PDF)

S6 Table Association of CFI with potential confounders. (PDF)

S7 Table Bias in one-sample MR. Estimation of the effect of X on Y for *β* = 0.8 by one-sample MR and CFMR, respectively. The simulations are detailed in Section 3.2.
(PDF)

S8 Table Cross-population CFMR estimates. Estimation of the effect of X on Y for *β* = 1 by cross-population CFMR (cpCFMR) and CFMR, respectively. The simulations are detailed in Section 3.3. Each scenario has been simulated 1000 times.
(PDF)
