# Supplementary material for: Cross-fitted instrument: A blueprint for one-sample Mendelian randomization
Source: PLoS Comput Biol. 2022 Aug 29;18(8):e1010268. doi: 10.1371/journal.pcbi.1010268 (PMC9462731; doi:10.1371/journal.pcbi.1010268)
Supplement: S1 Appendix — (PDF) [file pcbi.1010268.s001.pdf]

1 2-fold CFMR

The first step in a 2-fold Cross-Fitting Mendelian randomization (CFMR) is the random partitioning of the original sample into two samples of equal size, *sample 1* and *sample 2* (see Step 2 in Fig 1B)). After running a GWAS on each of these samples ($GWAS_1$ and $GWAS_2$) and clumping the results, SNPs that are suitable for use as IVs are selected and two sets of independent SNPs are created, set_1 and set_2. Using *sample 1* and set_1, we build a predictor ($pred_1$) of the exposure. Similarly, using *sample 2* and set_2, we build a predictor ($pred_2$) of the exposure. Next, we use $pred_1$ to predict the exposure in *sample 2*. Using the predicted exposure as instrument in *sample 2*, we estimate the causal effect of the exposure on the outcome in *sample 2* by performing a 2SLS. In a similar and complementary fashion, we estimate the causal effect of the exposure on the outcome in *sample 1* by performing a 2SLS using the predicted exposure in *sample 1* by $pred_2$ as instrument. The two estimates are referred to as $\hat{\beta}_0^2$ and $\hat{\beta}_0^1$, respectively. In the current context, CFI refers to the use of the predicted exposure of $pred_2$ as instrument for *sample 1*, and vice versa (see Fig 1 in the main text for a schematic overview).

2 Cross-population CFMR

Let us consider a dataset of N individuals comprising T heterogeneous populations. Each population t has n_t individuals within the dataset and $\sum_{t=1}^{T} n_t = N$. For simplicity, suppose that for each $t \in [1:T], n_t = \frac{N}{T}$. The populations can differ with respect to the minor allele frequency of a given variant and/or the overall genetic architecture [?]. Similarly, as in the Section **Setup** in the manuscript, let Y be a continuous outcome, X a continuous exposure, and Z a matrix containing Υ instruments. We assume that Y, X and Z are connected through the following linear regression models:

$$Y = \beta_0 X + U, \qquad \mathbb{E}[U|\Pi, Z] = 0 \tag{1}$$

$$X = Z\Pi + V, \qquad \mathbb{E}[V|Z] = 0 \tag{2}$$

The parameter of interest, β_0, is the causal effect of X on Y, Π is the vector of regression coefficients for the instruments, U and V are two correlated error terms, and $\mathbb{E}$ is the expectation operator. We assume that Z explains a proportion of the variance of X and that it can differ across populations. Thus, the vector Π and the minor allele of the variants used as instruments are allowed to differ across populations.

2.1 K-fold cross populations CFI

Briefly, a K-fold cross-population CFI consists of building a K-fold CFI for each population (as described in the Section **K-fold CFI** in the main text) and then concatenating them. More precisely, we define a K-fold cross-population CFI in the following manner: we split the sample into T sub-samples, with each sub-sample containing all the individuals of a given population within the original dataset. For each population t, we denote the set indices of the individuals within population t as I_t. We then partition I_t into a K-fold partition, where each partition is of size $\frac{n_t}{K}$. We refer to the K-fold partition of population t as $(I_{t,k})_{k \in 1:K}$. For a given population t, and for each k, we select $\Upsilon_{t,k}$ independent variants $\tilde{Z}_{t,k} = \left(Z_{1,t,k}, ..., Z_{\Upsilon_{t,k},t,k}\right)$ by performing a GWAS of X using the data in $I_{t,k}^c$. We then use these $n_{t,k}$ variants to build $pred_{t,k}$ (a predictor of X) and use the data with an index in $I_{t,k}^c$ as a training set. We then define the kth CFI in population t as:

$$\hat{X}_{t,k,i} = pred_{t,k}((Z_{i,1,t,k}, ..., Z_{i,\Upsilon_{t,k},t,k})_{i \in I_{t,k}}) \tag{3}$$

where d $Z_{i,l,t,k}$ is the variant $Z_{l,k}$ of individual i in population t. A K-fold cross-population CFI is defined as follows:

$$\check{X}_i = \hat{X}_{t,k,i} \text{ for } i \in I_{t,k} \tag{4}$$

The corresponding cross-population CFMR1 estimators are as follows:

$$\hat{\beta}_0 = \frac{1}{T \times K} \sum_{t=1}^{T} \sum_{k=1}^{K} 2SLS(X_{I_{t,k}}, Y_{I_{t,k}}, \hat{X}_{t,k}) \tag{5}$$

where $2SLS$ is the 2SLS estimator as defined in equation (6) in the main text.
This estimate corresponds to Step 5 in Fig 2B in the main text. A cross-population CFMR1 consists of performing an IVR on the complementary partition for each population. We then average the estimates of these IVRs to obtain the final estimate.

The cross-population CFMR2 estimate of β_0 is simply defined as:

$$\hat{\beta}_0 = 2SLS(X, Y, \check{X}) \tag{6}$$

Hence, CFMR2 consists of performing a single IVR on the entire dataset using the cross-population CFI $\hat{X}$ as instrument.

3 Simulations

3.1 Assessment of the statistical properties of CFMR

We assessed the behavior of CFMR in terms of the type I error, bias, convergence speed, and statistical power when LASSO is used to build the exposure predictors $pred_1$ and $pred_2$. We refer to CFMR as the estimator CFMR2. As explained in the main text, we chose CFMR2 over CFMR1 because, as is the case with double machine learning 1 and 2 (DML1 and DML2) described by Chernozhukov *et al.* [1], CFMR2 exhibits a better finite sample size performance than CFMR1 (for additional details, see [1]). Similar to the simulation setup in Deng *et al.* [2], we consider a set of 300 independent variants $(V_1, ..., V_{300})$ for each simulation, where each variant has a minor allele frequency of 0.3 and only the first five variants are associated with the exposure. Note that the only difference between our simulations and those of Deng *et al.* [2] is that the five variants associated with the exposure are assumed to be known in their simulations. Whereas Deng *et al.* only use the five truly-associated variants as instrument, we consider a more stringent setup where we purposefully dilute the effects of the five truly-associated variants by adding 295 non-associated variants. This setup makes it more challenging to construct accurate predictors of the exposure, particularly when the sample size is small and the IV is weak.

The exposure is generated as $X = \sum_{l=1}^{5} \pi_l V_l + v$ and the outcome is generated as $Y = \beta_0 X + u$, where v and u are two correlated error terms generated from a bivariate normal distribution.

$$\begin{pmatrix} \mathcal{U} \\ \mathcal{V} \end{pmatrix} \sim N \left[\begin{pmatrix} 0 \\ 0 \end{pmatrix}, \begin{pmatrix} 5 & 4.2 \\ 4.2 & 5 \end{pmatrix} \right]$$

The variants have the same effect (i.e. $\pi_1 = ... = \pi_5 = \pi$) where π is selected to ensure that the variants explain $h^2 = 20\%$ of the variation in the exposure.

Next, we applied CMFR to each simulated dataset using a LASSO-based IV after applying ten random splits. We considered various sample sizes (N = $1,000$ to $10,000$) and different β_0 values (-0.08, -0.05, 0, 0.05, and 0.08), similar to the simulations by Deng *et al.* [2]. For each combination of sample and effect size, we simulated 1000 datasets. Fig 1 summarizes the results of our simulations; we also provide a numerical summary of these simulations in Tables 1 and 2. It is clear from Fig 1 that CFMR and two-sample MR have very similar power. CFMR also shows excellent control of the type I error for the different nominal levels tested (see Table 1 and Fig 4).

We also assessed the type I error, bias, and power of CFMR for different estimates of the variance explained by the exposure (h^2 = 0%, 0.001%, 0.01%, 0.1%, 1%, 5%, and 10%) and different sample sizes (N = $1,000$, $5,000$, $10,000$, $50,000$, $100,000$, and $500,000$). For large particularly sample sizes (N= $100,000$ or N=$500,000$), however, we were unable to perform as many simulation as for smaller samples. Simulations were performed on a computer cluster with 32 CPUs and 128 GB RAM.

3.2 Comparison with one-sample MR

In this section, we perform a number of simulations to show that, under some settings where one-sample MR is heavily biased, CFMR remains conservative. The simulations were performed as follows. For each simulation, we considered a set of 300 independent variants $(V_1, ..., V_{300})$, where each variant has a minor allele frequency of 0.3 and only the first five variants are associated with the exposure. As mentioned in Section 3.1 above, these criteria are similar to the simulation setup in Deng et al. [2]. The exposure is generated as $X = \sum_{l=1}^{5} \pi V_l + v$ and the outcome as $Y = \beta_0 X + u$, where v and u are two correlated error terms generated from a bivariate normal distribution.

$$\begin{pmatrix} \mathcal{U} \\ \mathcal{V} \end{pmatrix} \sim N \left[\begin{pmatrix} 0 \\ 0 \end{pmatrix}, \begin{pmatrix} 5 & 4.9 \\ 4.9 & 5 \end{pmatrix} \right]$$

We consider the following two scenarios: 1) the variants explain 10% of the variance of X ($h^2 = 10\%$), and 2) the variants explain 20% of the variance of X ($h^2 = 20\%$). On each simulated dataset, we apply CFMR using a LASSO-based IV and ten random splits. We also build a predictor $pred_{all}$ of X using LASSO and the

entire dataset. We then use the prediction $pred_{all}$ on the entire data as instrument. We refer to 'one-sample MR estimates' when estimating the effect of X on Y using the prediction of $pred_{all}$. We consider various sample sizes, ranging from $1,000$ to $50,000$, and $\beta_0 = 0.08$. For each combination of sample and effect size, we simulate 1000 datasets. The results of these simulations are summarized in S7 Table and S32 Fig.

3.3 Two sub-populations

In this section, we perform a set of simulations to show that using cross-population CFMR2 allows a more accurate estimation of the causal effect than CFMR2 alone when dealing with multiple sub-populations. Similar to the previous section, we consider the following setup. For each simulation, we consider a set of 300 independent variants $(V_1, ..., V_{300})$. Here, we consider the setup where different sub-populations can have different genetic backgrounds for a given exposure, such as color blindness which is more heritable in men than women [**?**].

In sub-population 1, the exposure is generated as $X = \sum_{l=1}^{10} \pi_{1,l} V_l + v$ and the outcome as $Y = \beta_0 X + u$, where v and u are two correlated error terms generated from a bivariate normal distribution with the covariance as the bivariate normal distribution used in the Section **Comparison with one-sample MR** in the main text. The coefficients $\pi_{1,l}$ are scaled to ensure that $(V_1, ..., V_{10})$ explain 20% of the variance of X.

In sub-population 2, the exposure is generated as $X = \sum_{l=5}^{15} \pi_{2,l} V_l + v$ and the outcome as $Y = \beta_0 X + u$, where v and u are two correlated error terms generated from a bivariate normal distribution with the covariance as the bivariate normal distribution used in the Section **Comparison with one-sample MR** in the main text. The coefficients $\pi_{2,l}$ are scaled to ensure that $(V_1, ..., V_{10})$ explain 10% of the variance of X. Thus, sub-populations 1 and 2 share 50% of the genetic architecture of the exposure. However, the coefficients of the variants that affect the exposure in both sub-populations have different effects across the two populations. This is because both the heritability and the minor allele frequency differs between sub-populations 1 and 2.

For each simulation, we applied CFMR2 and cross-population CFMR2 using a LASSO-based IV and ten random splits. We considered various sample sizes, ranging from $1,000$ to $50,000$, and set $\beta_0 = 1$. For each combination of sample and effect size, we simulated 1000 datasets. The results are summarized in S8 Table 8 and S33 Fig.

4 Study description

The Norwegian Mother, Father, and Child Cohort Study (MoBa) is an ongoing nationwide pregnancy cohort [3,4]. Participants in MoBa were enrolled in the study between 1999 and 2008 from 50 of the 52 hospitals in Norway. The vast majority of MoBa participants are of Caucasian origin. The genotypes in the MoBa dataset were obtained from array-based genotyping of whole-blood DNA from parents and umbilical-cord blood DNA from newborns [**?**]. We excluded stillbirths, twins, and children with missing data in the Medical Birth Registry of Norway (MBRN). Pre-pregnancy maternal BMI was calculated on the basis of self-reported height and weight. Information on the children's birth weight was extracted from medical records.

Approximately $30,000$ mother-father-newborn trios in the MoBa dataset were genotyped using the Illumina HumanCoreExome BeadChip (San Diego, CA, USA) housing more than 240,000 probes. We removed ethnic outliers based on visual checks using the first three principal components. We used the Haplotype Reference Consortium (HRC) reference data, version HRC.r1.1 (http://www.haplotype-reference-consortium.org/), for imputation of additional genotypes in the MoBa dataset. The imputation itself was performed using the free genotype imputation and phasing service of the Sanger Imputation Server (https://imputation.sanger.ac.uk/).

Imputation quality was assessed by (i) hard-calling markers with an INFO quality score greater than 0.7, (ii) testing for Mendelian inconsistencies, excess of heterozygosity, and significant deviation from Hardy-Weinberg equilibrium (HWE), and (iii) screening for high rates of missingness. The remaining set of $7,947,894$ SNPs met the following criteria and were included in the 10 GWASes performed in the current CFMR: (i) call rate $\geq 98\%$, (ii) minor allele frequency (MAF) $\geq 1\%$, and (iii) HWE test P-value $\geq 10^4$. Samples with a call rate $\leq 98\%$ and an excess heterozygosity $\geq 4SD$ were excluded. Finally, 1647 mother-newborn dyads characterized by one or more of the following features were excluded from the current analyses: multiple births, stillbirths, congenital anomalies, births before 37 weeks gestation, or pregnancy hypertension.

References

1. Victor Chernozhukov, Denis Chetverikov, Mert Demirer, Esther Duflo, Christian Hansen, Whitney Newey, et al. Double/debiased machine learning for treatment and structural parameters. *The Econometrics Journal*, 21(1):C1–C68, 2018. _eprint: https://onlinelibrary.wiley.com/doi/pdf/10.1111/ectj.12097.

2. Lu Deng, Han Zhang, and Kai Yu. Power calculation for the general two-sample Mendelian randomization analysis. *Genetic Epidemiology*, 44(3):290–299, 2020. _eprint: https://onlinelibrary.wiley.com/doi/pdf/10.1002/gepi.22284.

3. Per Magnus, Charlotte Birke, Kristine Vejrup, Anita Haugan, Elin Alsaker, Anne Kjersti Daltveit, Marte Handal et al. Cohort Profile Update: The Norwegian Mother and Child Cohort Study (MoBa). *International Journal of Epidemiology*, 45(2):382–388, 2016.

4. Liv Paltiel, Haugan Anita, Trine Skjerden, Kari Harbak, Siri Bækken, Stensrud Nina Kristin, et al. The biobank of the Norwegian Mother and Child Cohort Study present status. *Norsk Epidemiologi*, 24(1-2)
