# Supplementary figures and images for: Cross-fitted instrument: A blueprint for one-sample Mendelian randomization
Source: PLoS Comput Biol. 2022 Aug 29;18(8):e1010268. doi: 10.1371/journal.pcbi.1010268 (PMC9462731; doi:10.1371/journal.pcbi.1010268)

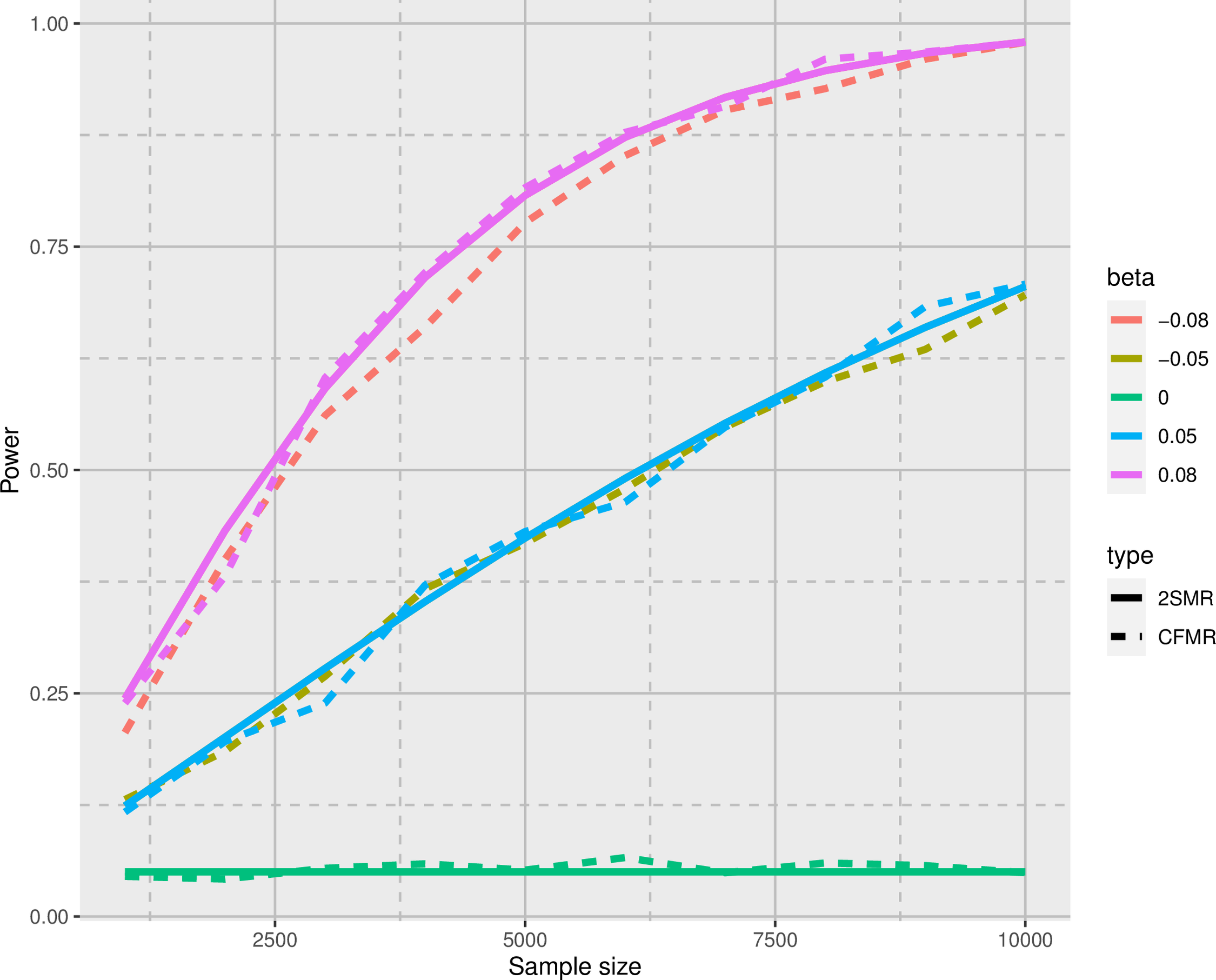

Supplement: S1 Fig — Power curves for CFMR versus two-sample MR (2SMR) using the simulation setup described in the Simulations section in the main text (with h2 = 20%). The dashed lines represent power curves for CFMR and the solid lines represent the theoretical power for 2SMR [24]. Note that the solid pink line covers the solid red line perfectly. These lines fully overlap as a result of symmetry and both lines correspond to the same effect size but have opposite signs. The same is the case for the solid blue and gold lines. (TIF) [file pcbi.1010268.s002.tif]

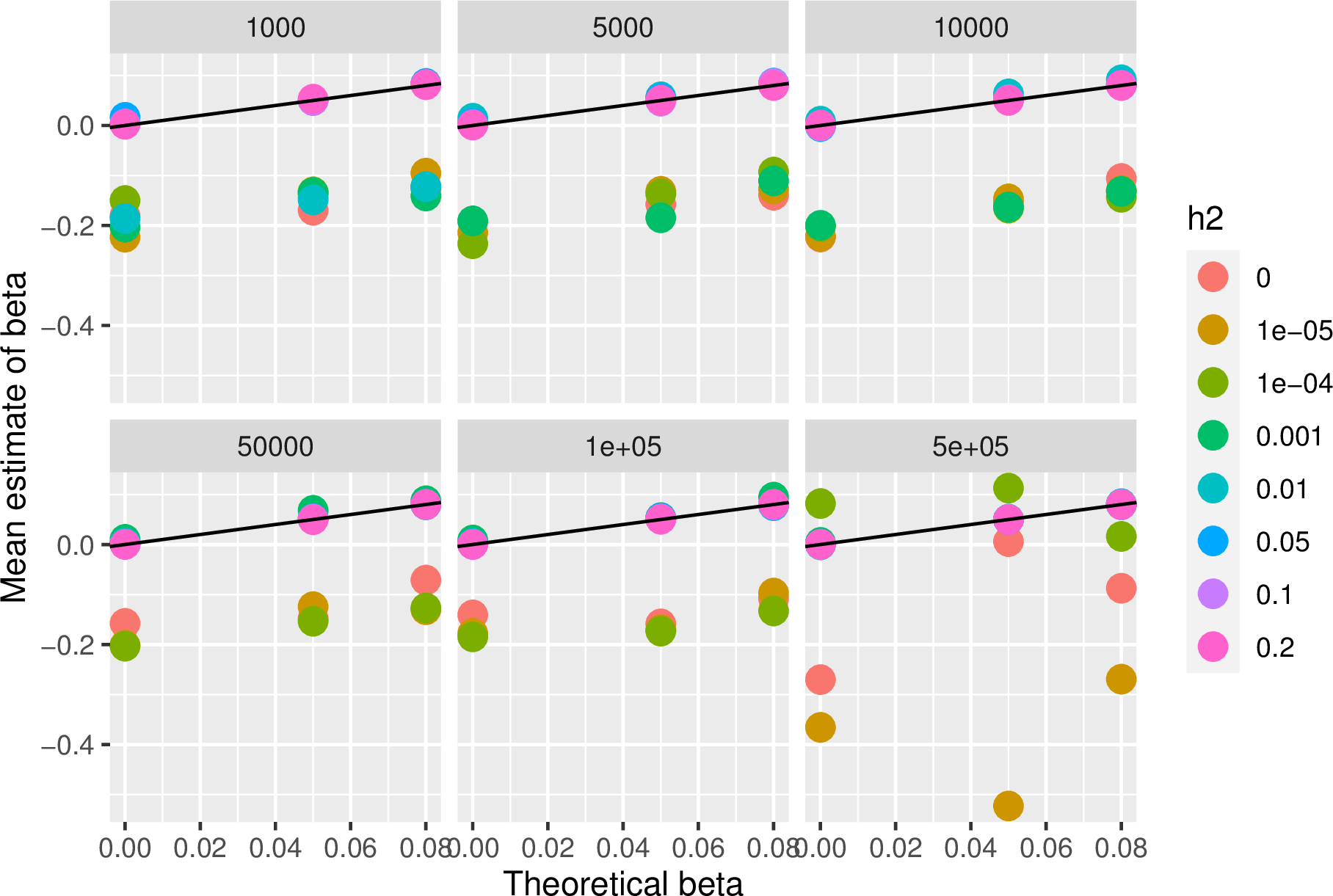

Supplement: S2 Fig — Mean estimate of beta by CFMR against the true beta for different values of beta, h2 and N. (TIF) [file pcbi.1010268.s003.tif]

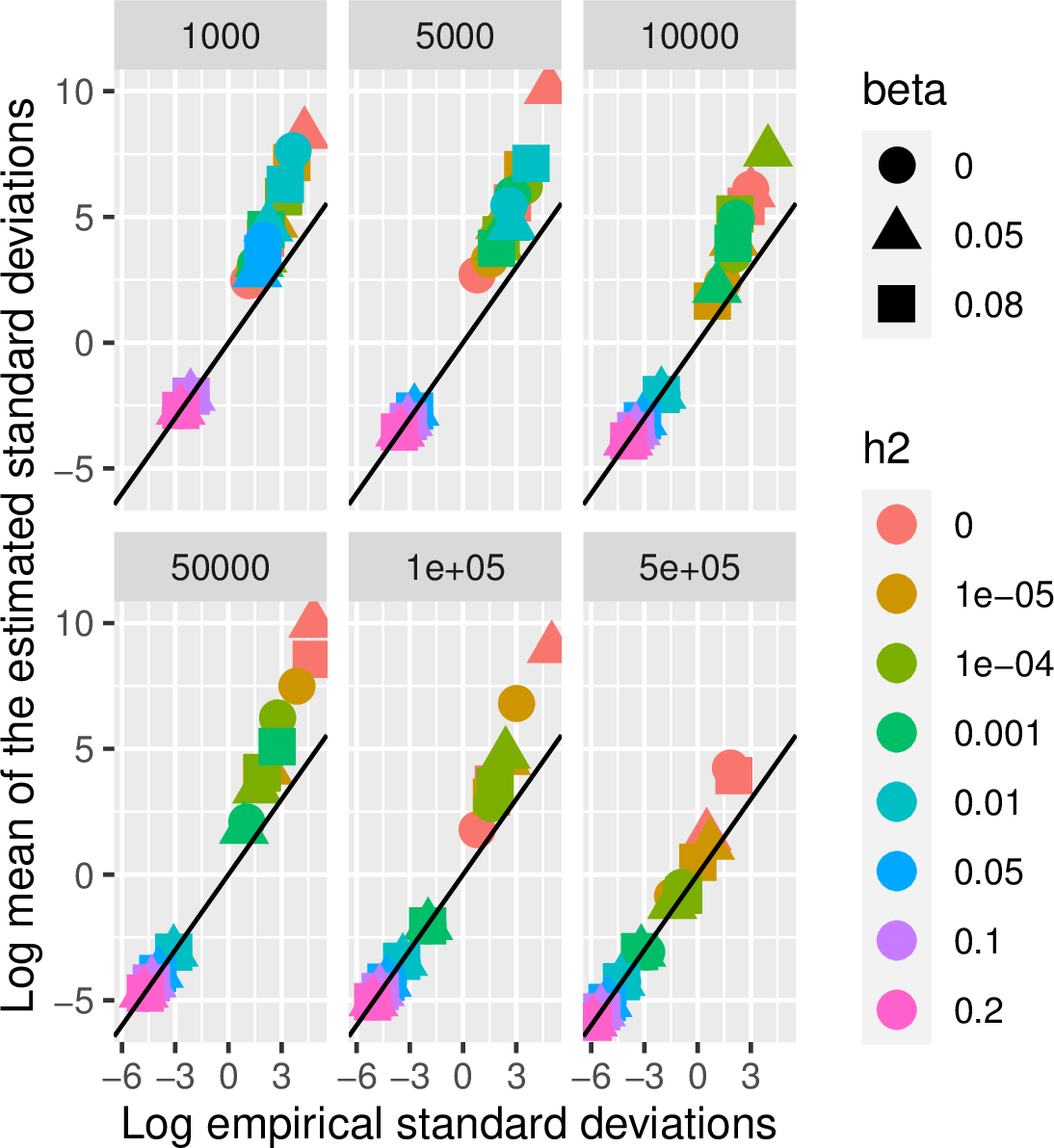

Supplement: S3 Fig — Empirical standard deviation of CFMR against the mean of the estimated standard deviations of CFMR for different values of beta, h2 and N. (TIF) [file pcbi.1010268.s004.tif]

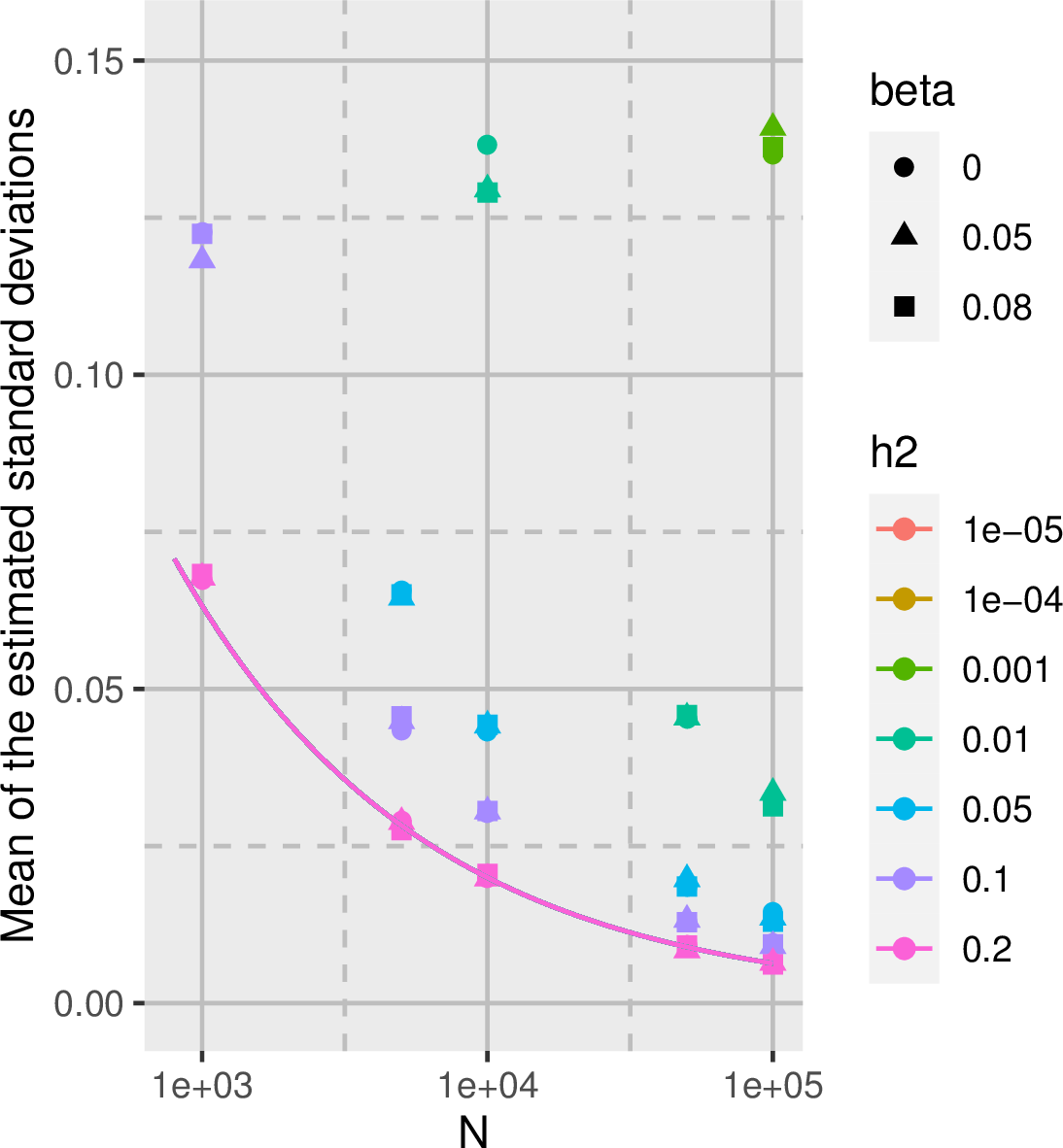

Supplement: S4 Fig — Mean of the estimated standard deviations of CFMR for different values of beta, h2 and N. Each configuration was simulated 1000 times. The solid line is the function f(x)=σsqrt(x), where σ2 is the variance of Υ in the simulations described in Section 3.1. (TIF) [file pcbi.1010268.s005.tif]

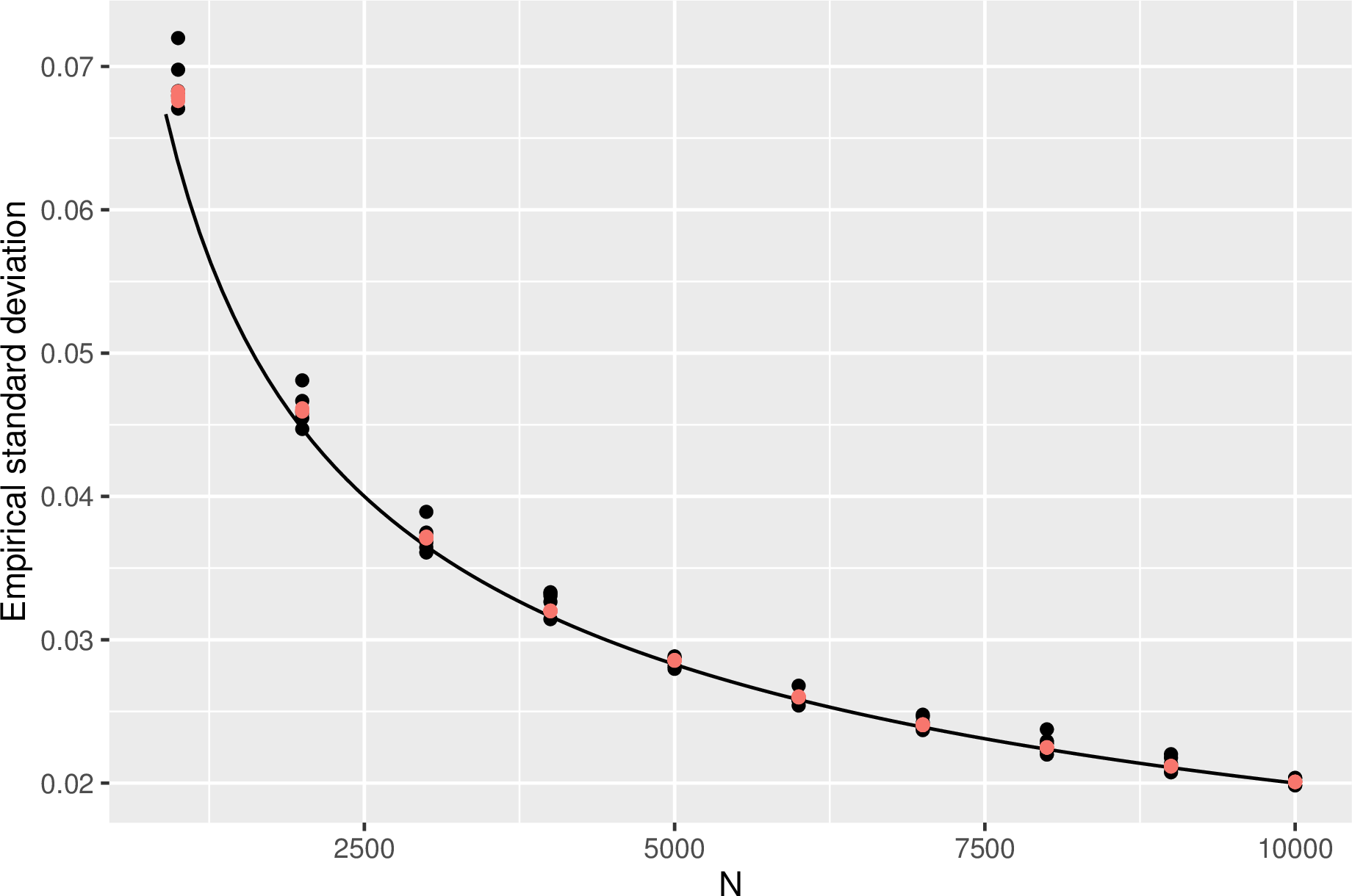

Supplement: S5 Fig — The black dots correspond to the empirical standard deviation of CFMR for various values of β0. The red dots correspond to the empirical standard deviation of CFMR for various values of β0 (h2 = 20%). The black line corresponds to the function f(x)=σsqrt(x), where σ2 is the variance of the simulations described in Section 3.1. (TIF) [file pcbi.1010268.s006.tif]

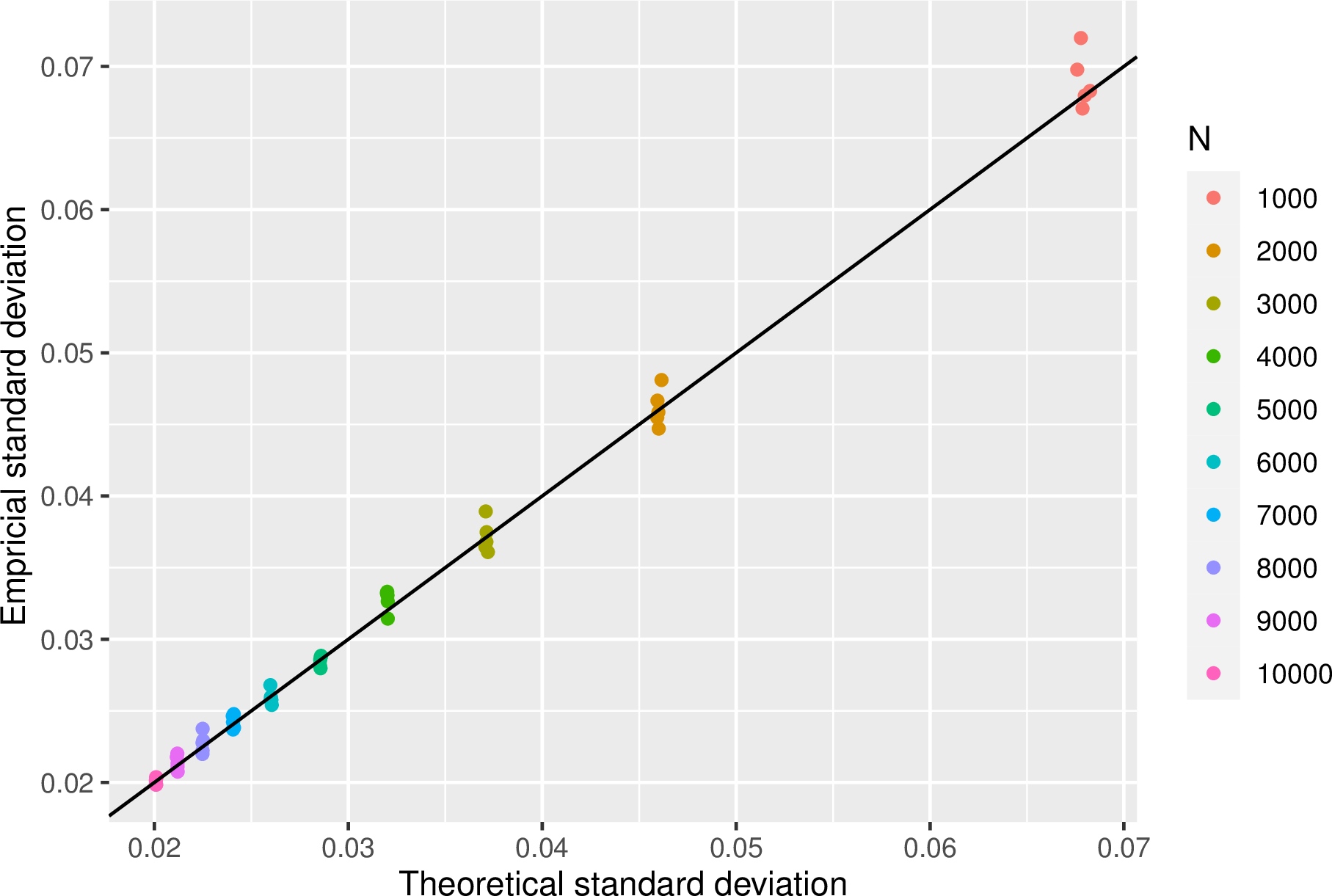

Supplement: S6 Fig — Empirical standard deviation of CFMR against its theoretical standard deviation for different values of β0 and N, with h2 = 20%. (TIF) [file pcbi.1010268.s007.tif]

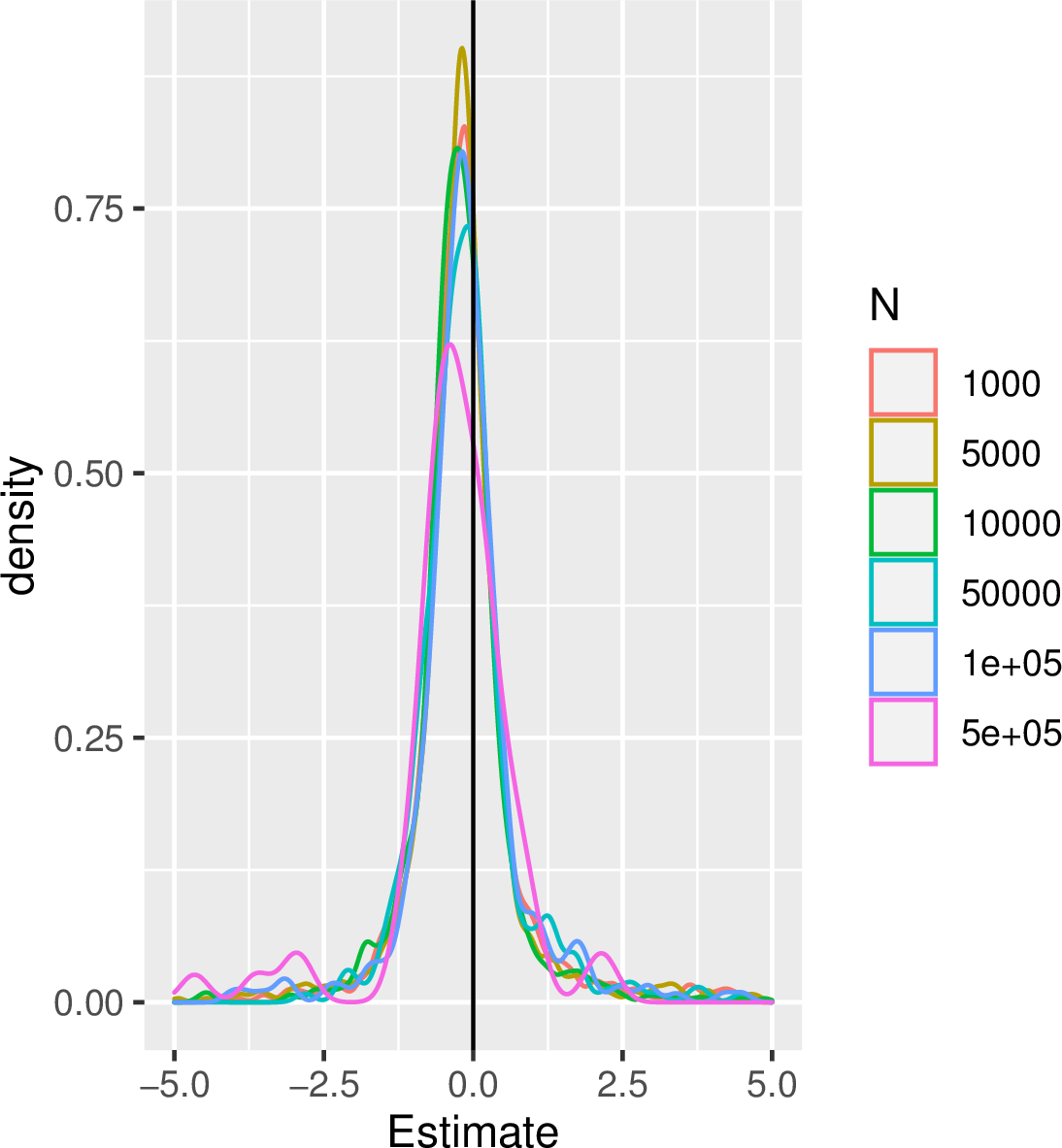

Supplement: S7 Fig — Density of the estimation of CFMR when no instrument is causally related to the exposure, based on different sample sizes. (TIF) [file pcbi.1010268.s008.tif]

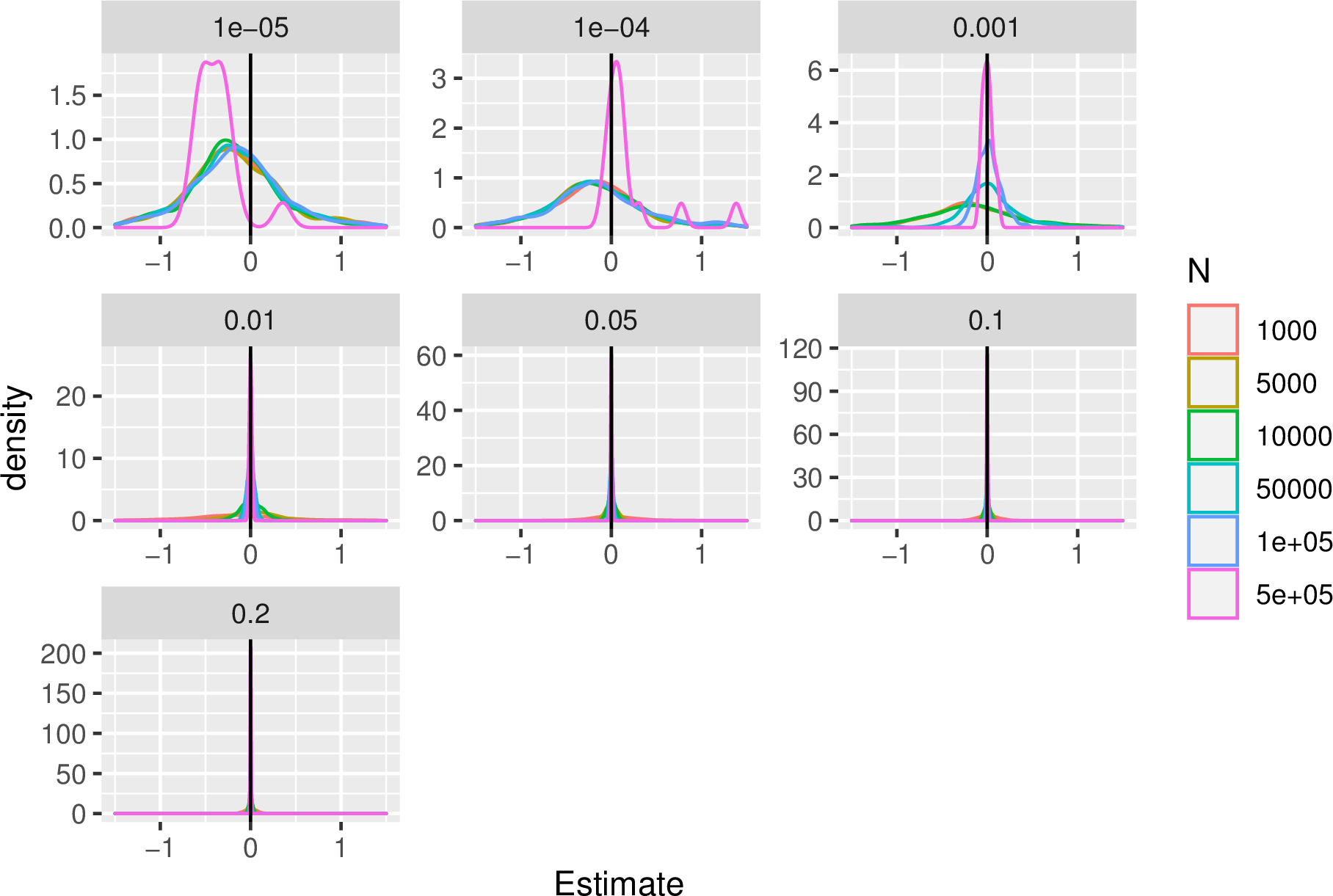

Supplement: S8 Fig — Density of the estimation of CFMR when β0 = 0 for different sample sizes. The variance explained by the instrument (h2) is displayed on top of each plot. (TIF) [file pcbi.1010268.s009.tif]

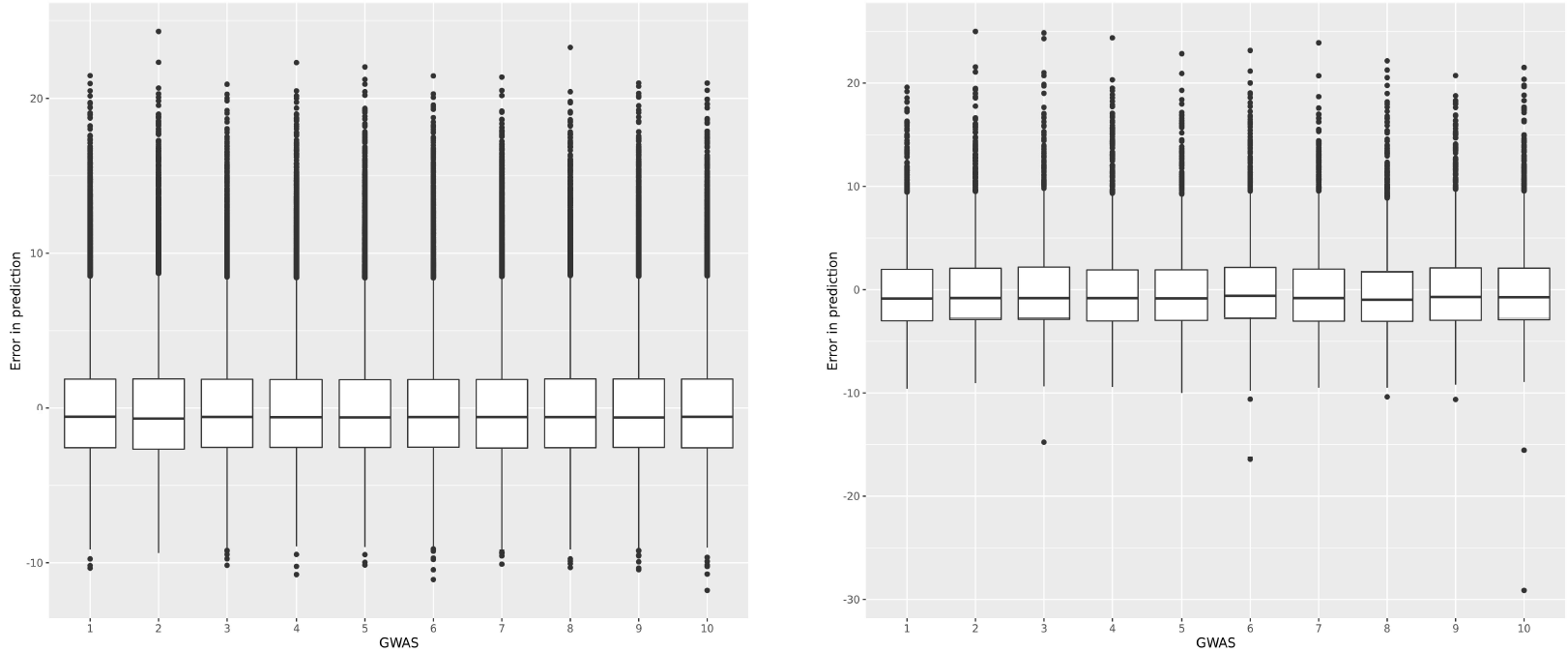

Supplement: S9 Fig — Predicted pre-pregnancy BMI performance on test and training sets. Left panel: the boxplot of the difference between the predicted pre-pregnancy BMI and the observed pre-pregnancy BMI on each training set, using a P-value threshold of 10−3. Right panel: the boxplot of the difference between the predicted pre-pregnancy BMI and the observed pre-pregnancy BMI on each test set, using a P-value threshold of 10−3. (TIF) [file pcbi.1010268.s010.tif]

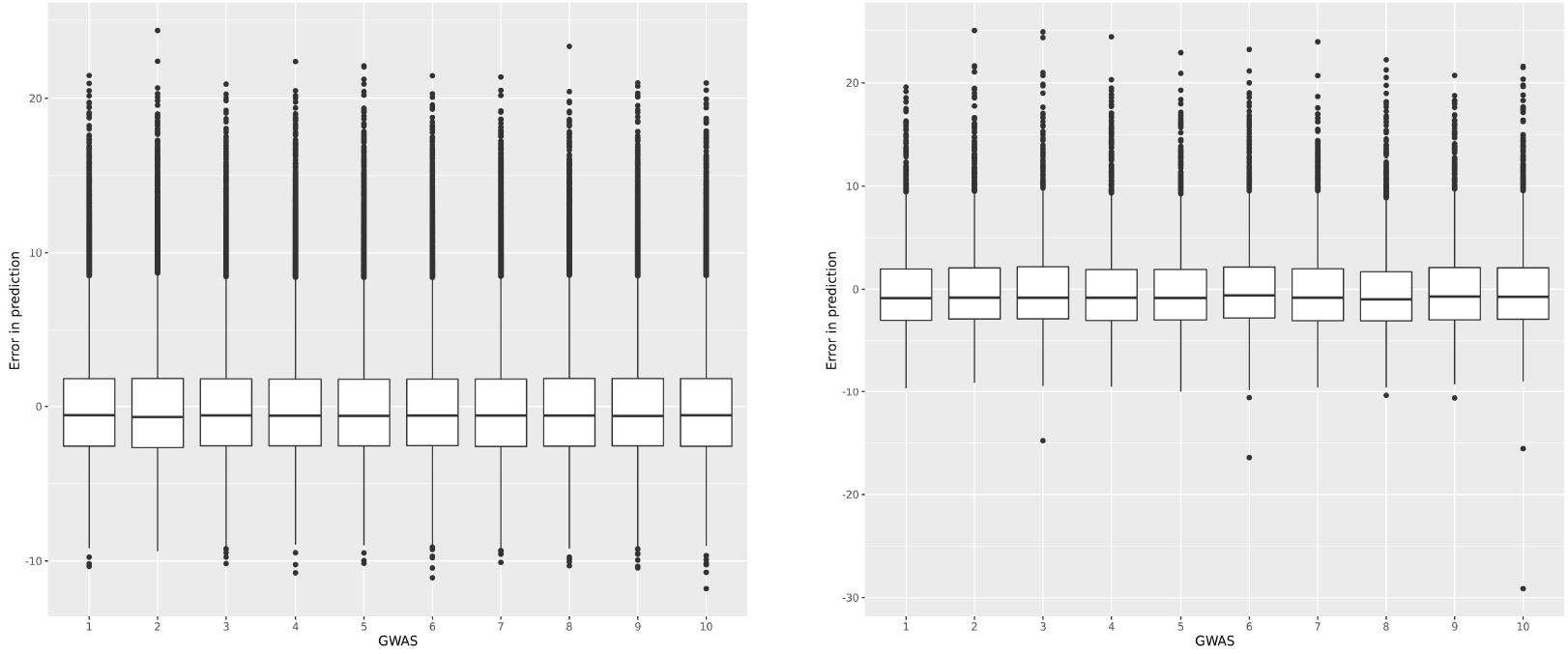

Supplement: S10 Fig — Predicted pre-pregnancy BMI performance on test and training sets. Left panel: the boxplot of the difference between the predicted pre-pregnancy BMI and the observed pre-pregnancy BMI on each training set, using a P-value threshold of 10−4. Right panel: the boxplot of the difference between the predicted pre-pregnancy BMI and the observed pre-pregnancy BMI on each test set, using a P-value threshold of 10−4. (TIF) [file pcbi.1010268.s011.tif]

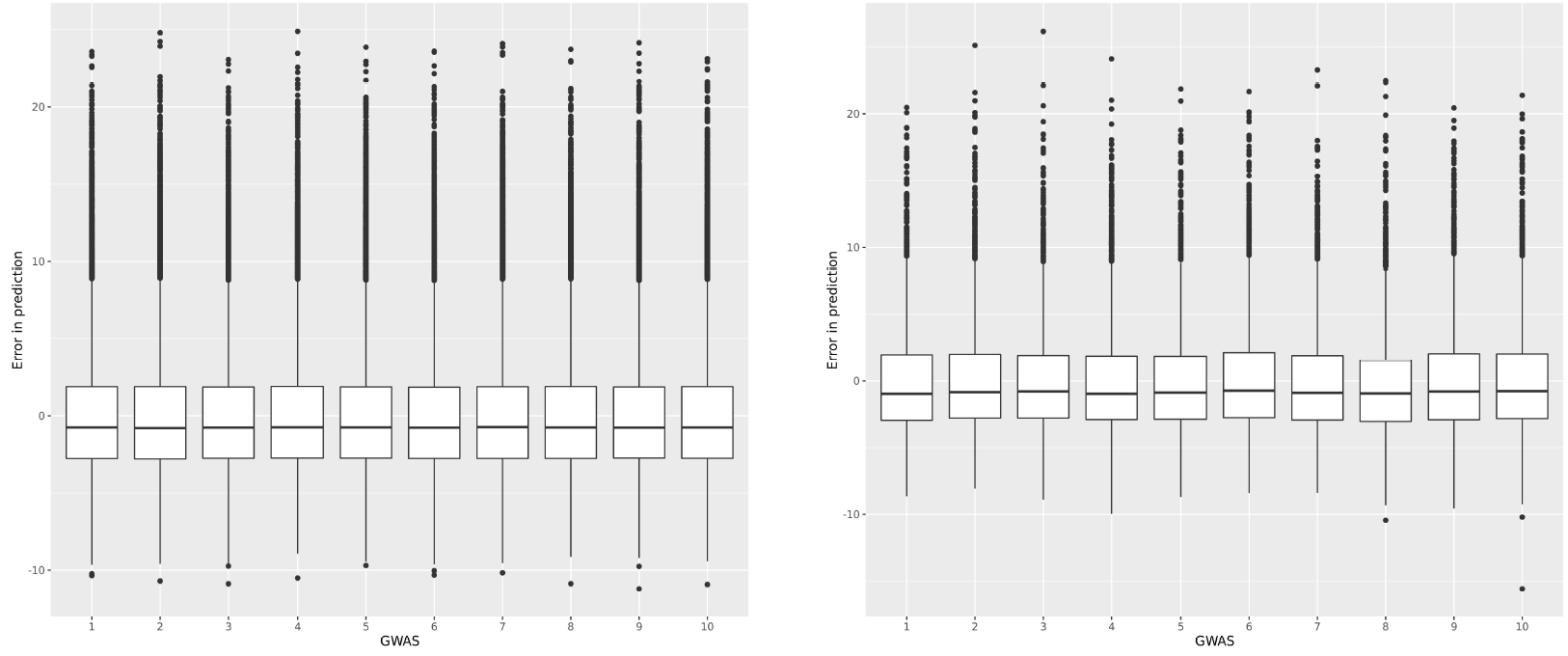

Supplement: S11 Fig — Predicted pre-pregnancy BMI performance on test and training sets. Left panel: the boxplot of the difference between the predicted pre-pregnancy BMI and the observed pre-pregnancy BMI on each training set, using a P-value threshold of 10−5. Right panel: the boxplot of the difference between the predicted pre-pregnancy BMI and the observed pre-pregnancy BMI on each test set, using a P-value threshold of 10−5. (TIF) [file pcbi.1010268.s012.tif]

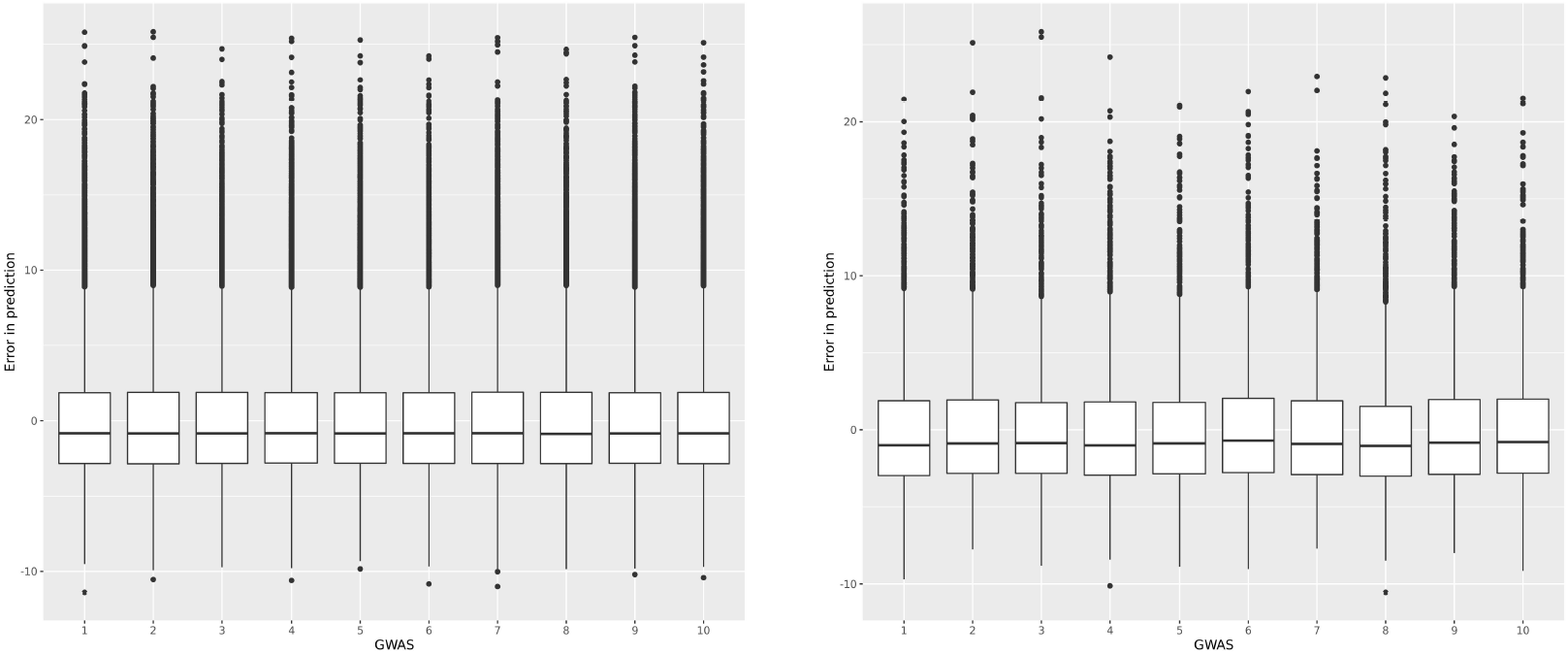

Supplement: S12 Fig — Predicted pre-pregnancy BMI performance on test and training sets. Left panel: the boxplot of the difference between the predicted pre-pregnancy BMI and the observed pre-pregnancy BMI on each training set, using a P-value threshold of 10−6. Right panel: the boxplot of the difference between the predicted pre-pregnancy BMI and the observed pre-pregnancy BMI on each test set, using a P-value threshold of 10−6. (TIF) [file pcbi.1010268.s013.tif]

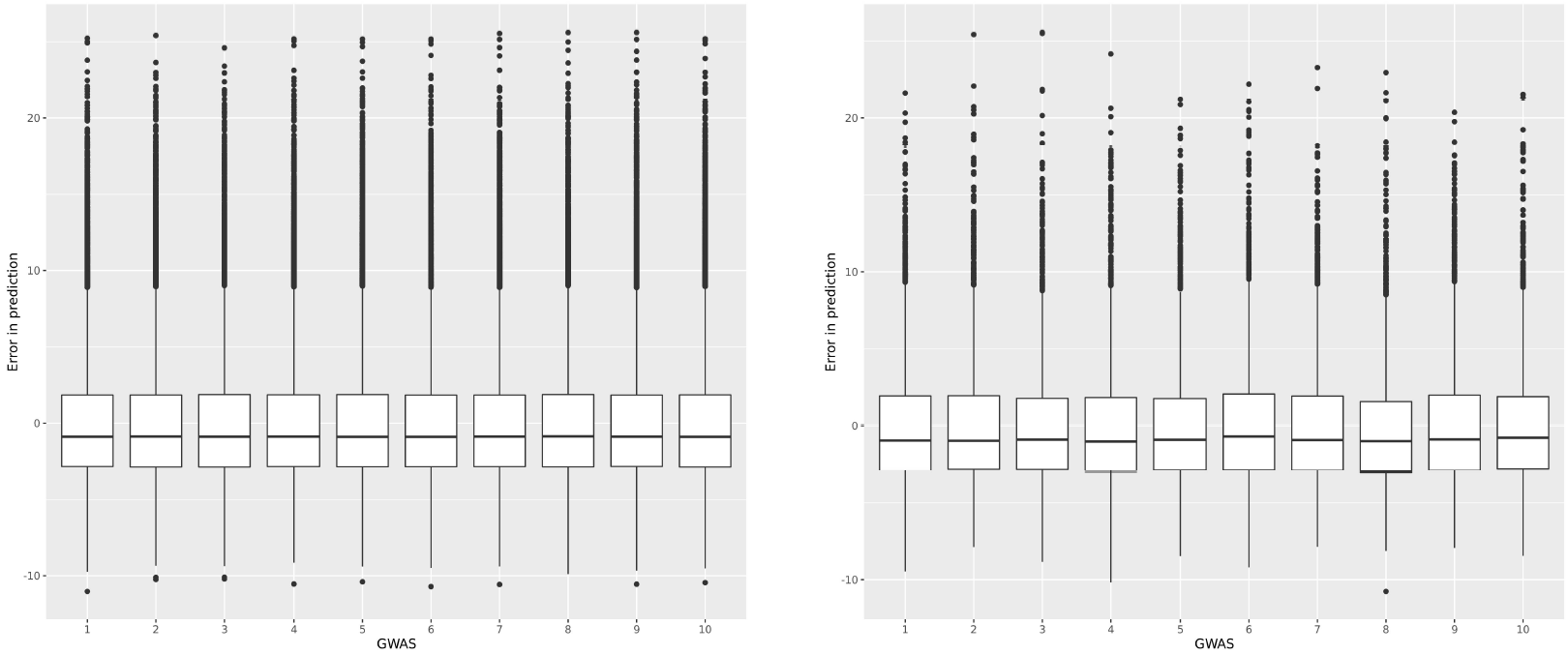

Supplement: S13 Fig — Predicted pre-pregnancy BMI performance on test and training sets. Left panel: the boxplot of the difference between the predicted pre-pregnancy BMI and the observed pre-pregnancy BMI on each training set, using a P-value threshold of 10−7. Right panel: the boxplot of the difference between the predicted pre-pregnancy BMI and the observed pre-pregnancy BMI on each test set, using a P-value threshold of 10−7. (TIF) [file pcbi.1010268.s014.tif]

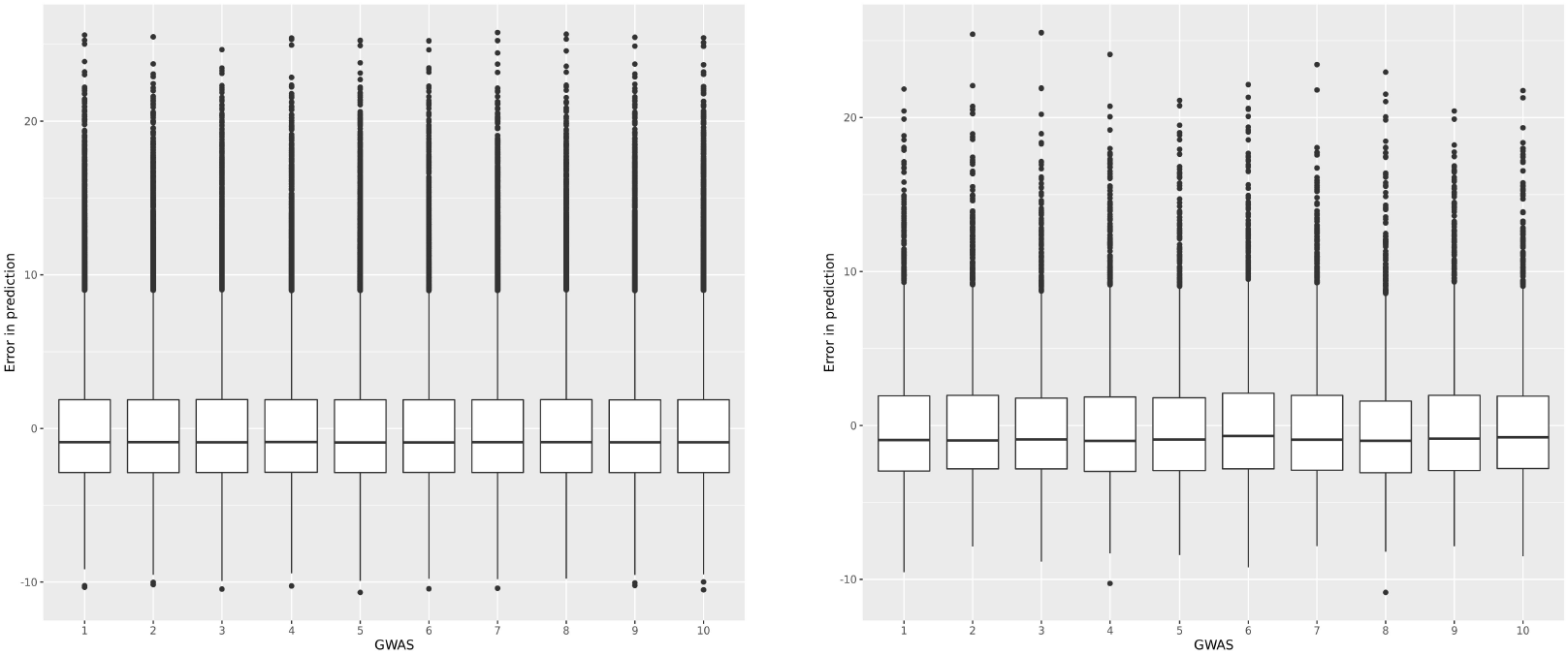

Supplement: S14 Fig — Predicted pre-pregnancy BMI performance on test and training sets. Left panel: the boxplot of the difference between the predicted pre-pregnancy BMI and the observed pre-pregnancy BMI on each training set, using a P-value threshold of 10−8. Right panel: the boxplot of the difference between the predicted pre-pregnancy BMI and the observed pre-pregnancy BMI on each test set, using a P-value threshold of 10−8. (TIF) [file pcbi.1010268.s015.tif]

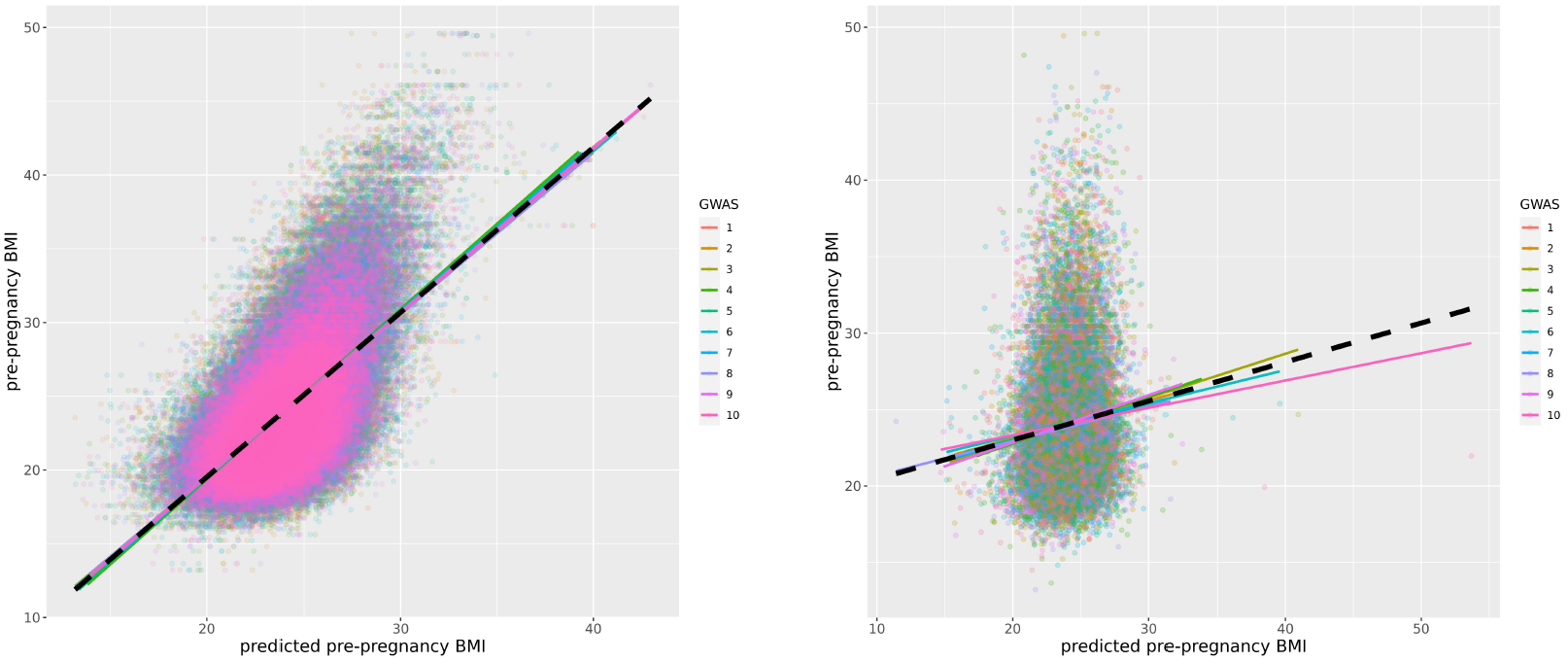

Supplement: S15 Fig — Predicted pre-pregnancy BMI performance on test and training sets. Left panel: the bivariate plot of the predicted pre-pregnancy BMI on training sets against true values using a P-value threshold of 10−3. Right panel: the bivariate plot of the predicted pre-pregnancy BMI on test sets against the true values using a P-value threshold of 10−3. (TIF) [file pcbi.1010268.s016.tif]

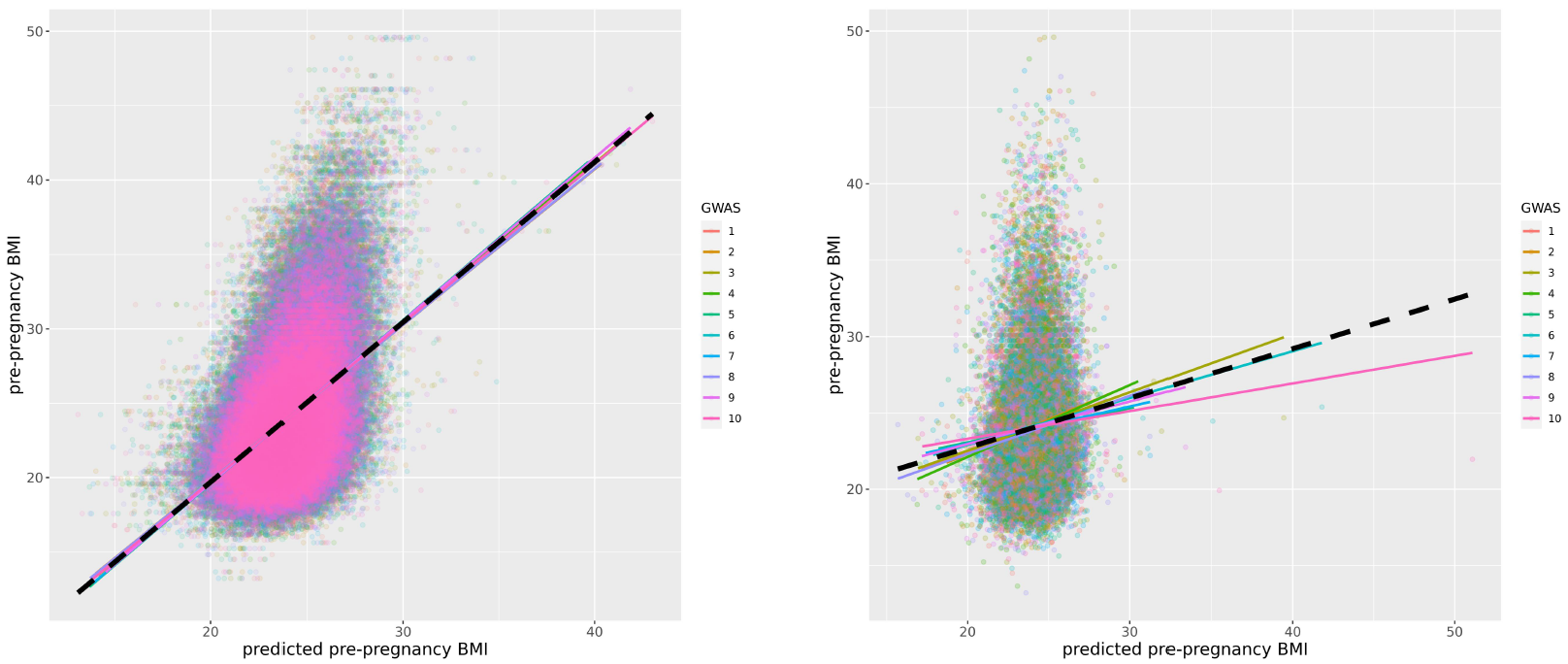

Supplement: S16 Fig — Predicted pre-pregnancy BMI performance on test and training sets. Left panel: the bivariate plot of the predicted pre-pregnancy BMI on training sets against true values using a P-value threshold of 10−4. Right panel: the bivariate plot of the predicted pre-pregnancy BMI on test sets against the true values using a P-value threshold of 10−4. (TIF) [file pcbi.1010268.s017.tif]

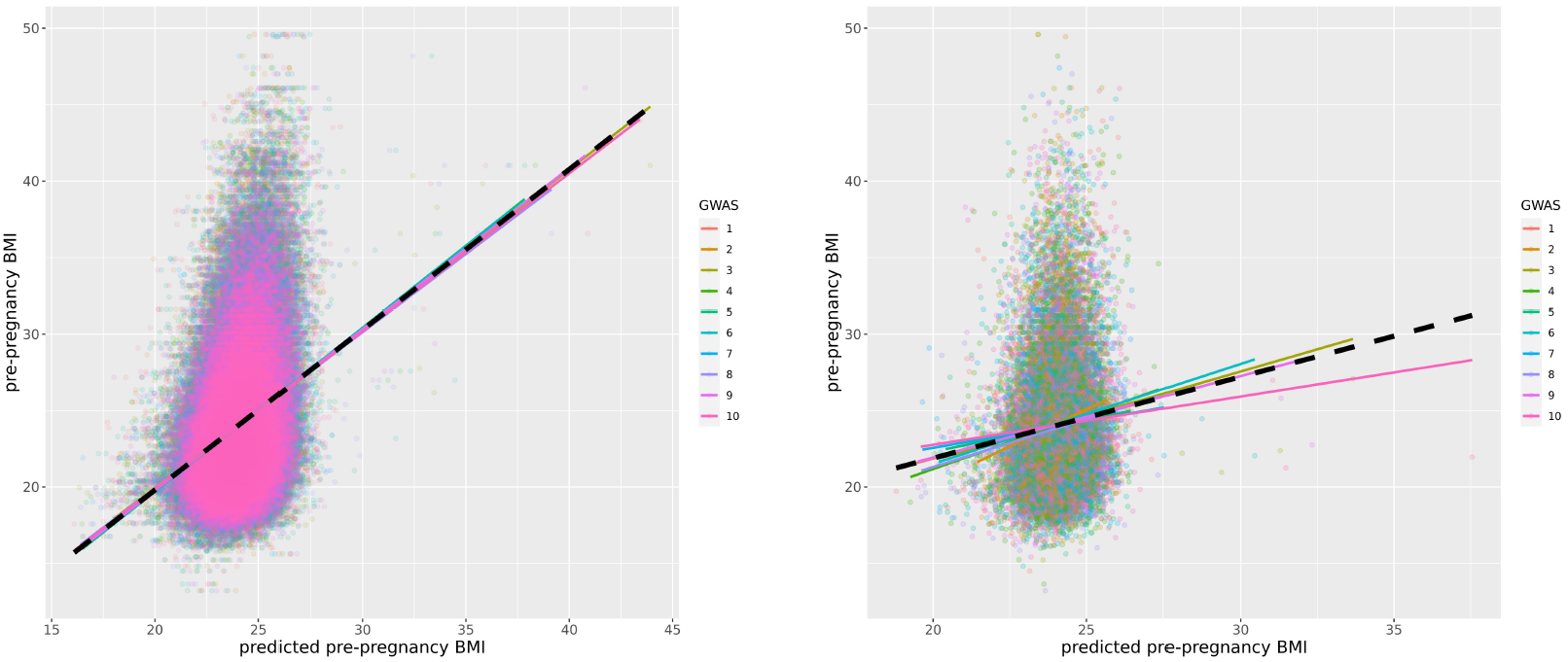

Supplement: S17 Fig — Predicted pre-pregnancy BMI performance on test and training sets. Left panel: the bivariate plot of the predicted pre-pregnancy BMI on training sets against true values using a P-value threshold of 10−5. Right panel: the bivariate plot of the predicted pre-pregnancy BMI on test sets against the true values using a P-value threshold of 10−5. (TIF) [file pcbi.1010268.s018.tif]

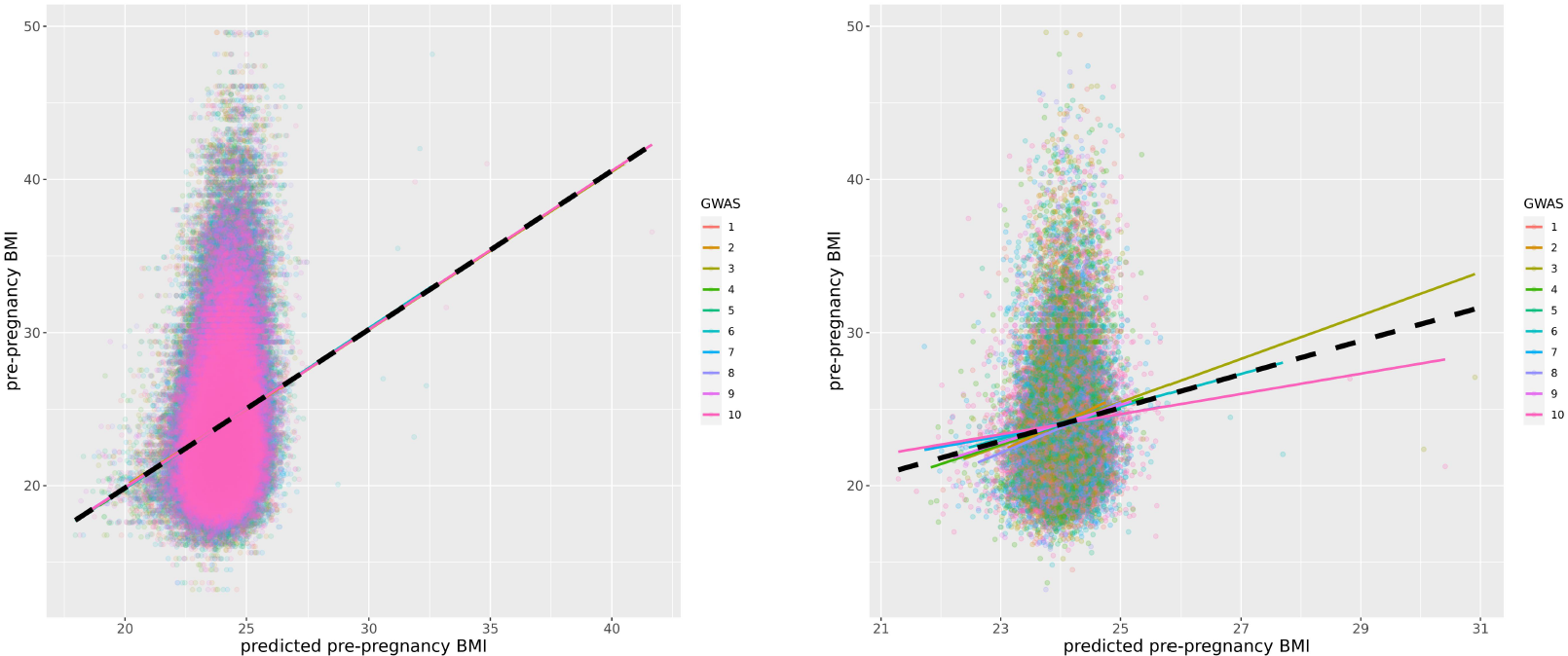

Supplement: S18 Fig — Predicted pre-pregnancy BMI performance on test and training sets. Left panel: the bivariate plot of the predicted pre-pregnancy BMI on training sets against true values using a P-value threshold of 10−6. Right panel: the bivariate plot of the predicted pre-pregnancy BMI on test sets against the true values using a P-value threshold of 10−6. (TIF) [file pcbi.1010268.s019.tif]

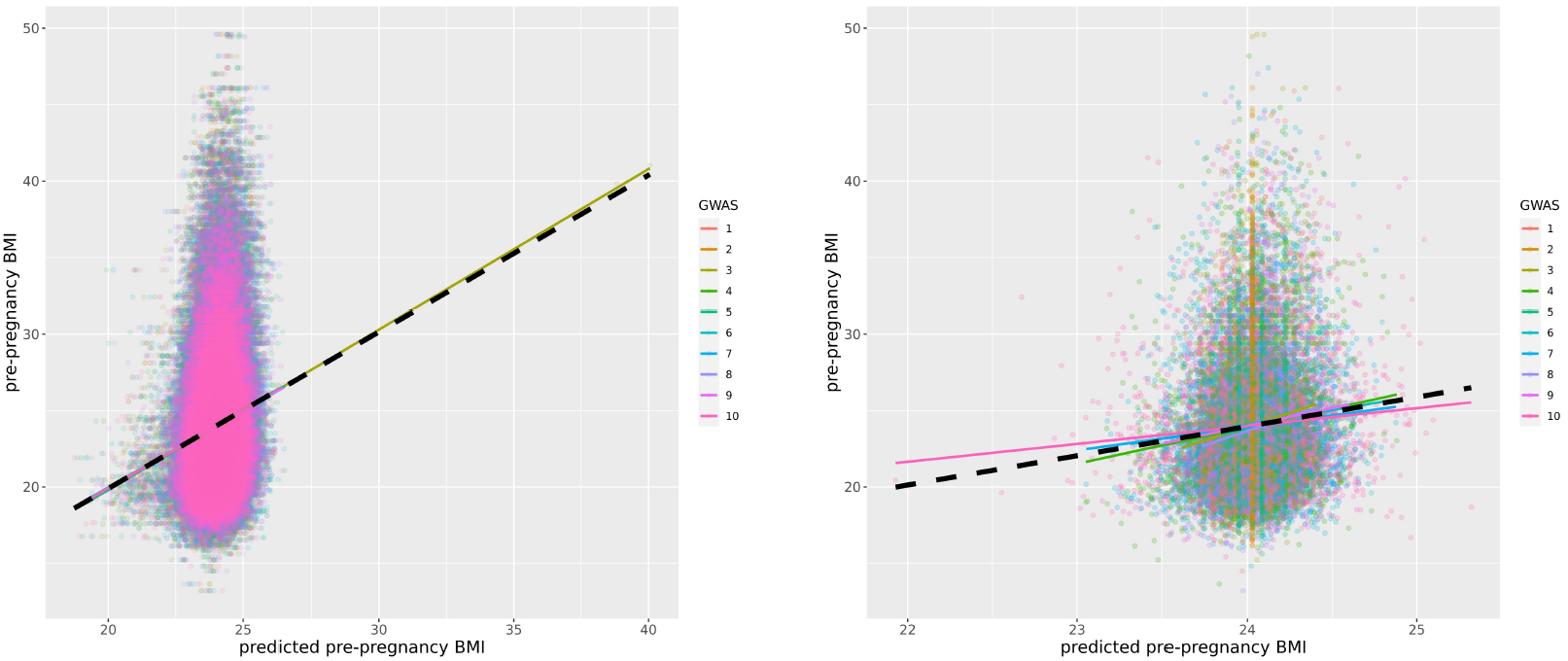

Supplement: S19 Fig — Predicted pre-pregnancy BMI performance on test and training sets. Left panel: the bivariate plot of the predicted pre-pregnancy BMI on training sets against true values using a P-value threshold of 10−7. Right panel: the bivariate plot of the predicted pre-pregnancy BMI on test sets against the true values using a P-value threshold of 10−7. (TIF) [file pcbi.1010268.s020.tif]

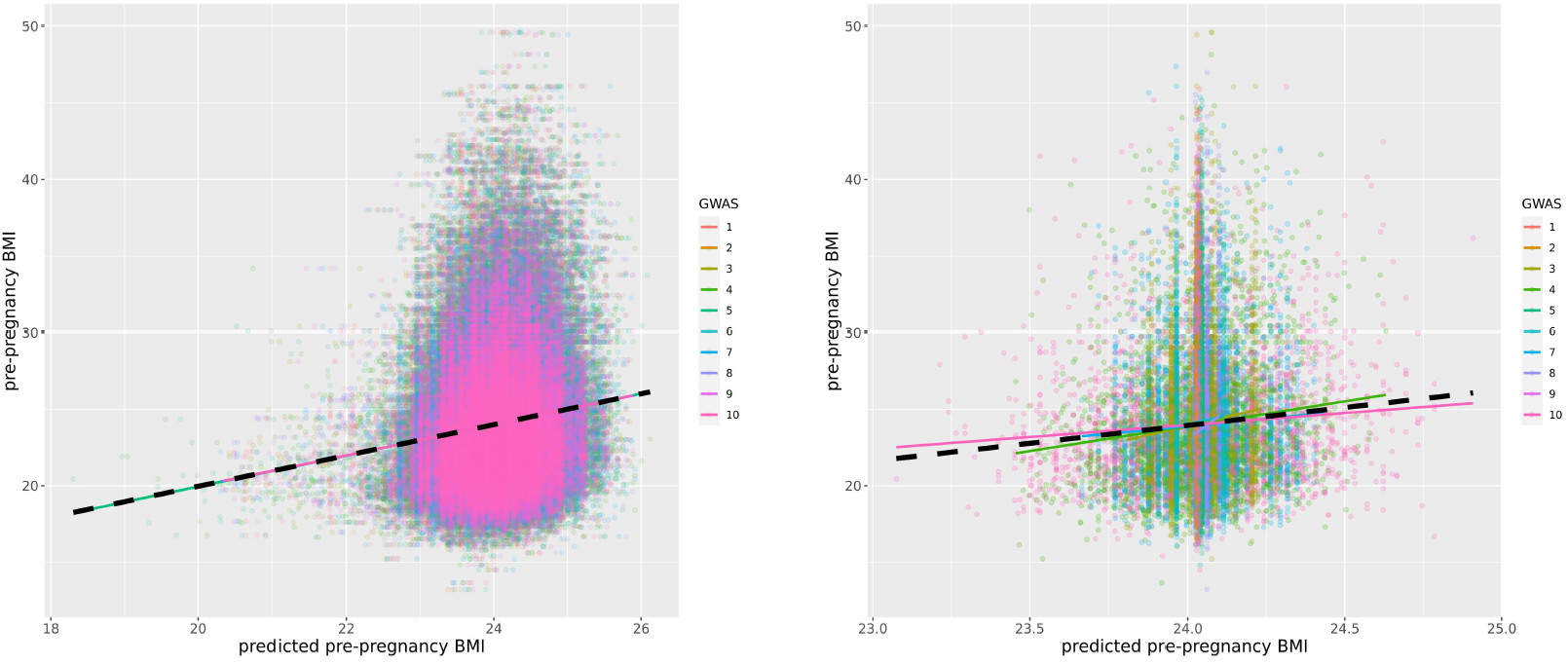

Supplement: S20 Fig — Predicted pre-pregnancy BMI performance on test and training sets. Left panel: the bivariate plot of the predicted pre-pregnancy BMI on training sets against true values using a P-value threshold of 10−8. Right panel: the bivariate plot of the predicted pre-pregnancy BMI on test sets against the true values using a P-value threshold of 10−8. (TIF) [file pcbi.1010268.s021.tif]

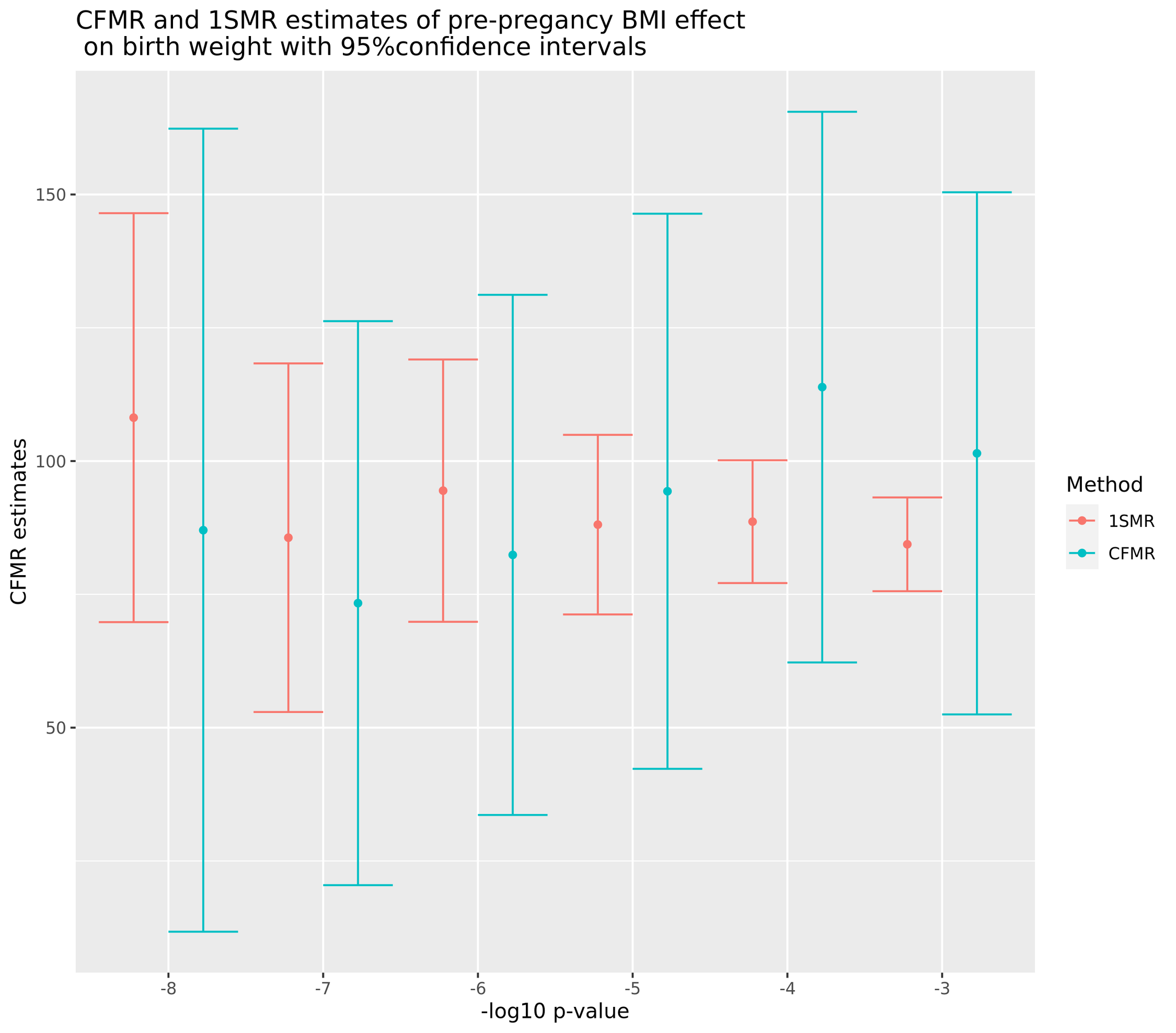

Supplement: S21 Fig — CFMR and one-sample MR (1SMR) estimates of the effect of pre-pregnancy maternal BMI on birth weight, with 95% confidence intervals. (TIF) [file pcbi.1010268.s022.tif]

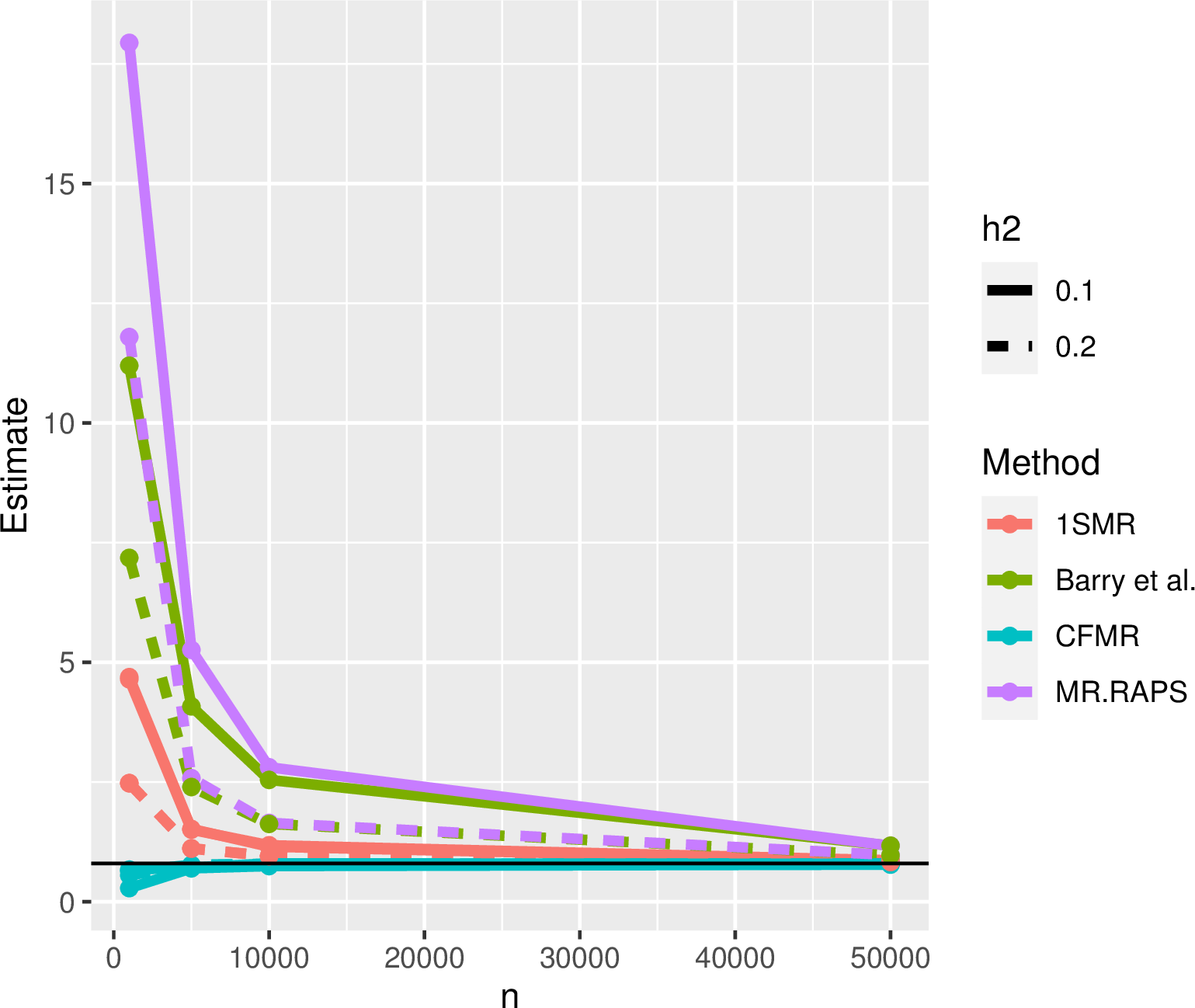

Supplement: S22 Fig — Summary of the simulations performed in Section 3.2. The x-axis corresponds to the number of individuals used in each simulation (1000; 5, 000; 10, 000; and 50, 000), and the y-axis corresponds to the estimated effect. The solid horizontal black line corresponds to the true value of the effect to be estimated. The dashed and solid lines lines correspond to the variance X explained by the genetic marker used as instrument (10% and 20%). The different types of lines correspond to the variance X explained by the genetic marker used as instruments (10% and 20%). The red lines correspond to the mean estimate using one-sample MR. The green lines correspond to the mean estimate using the Barry et al. method. The pink lines correspond to the mean estimate using MR RAPS and the blue lines correspond to the mean estimate using CFMR. (TIF) [file pcbi.1010268.s023.tif]

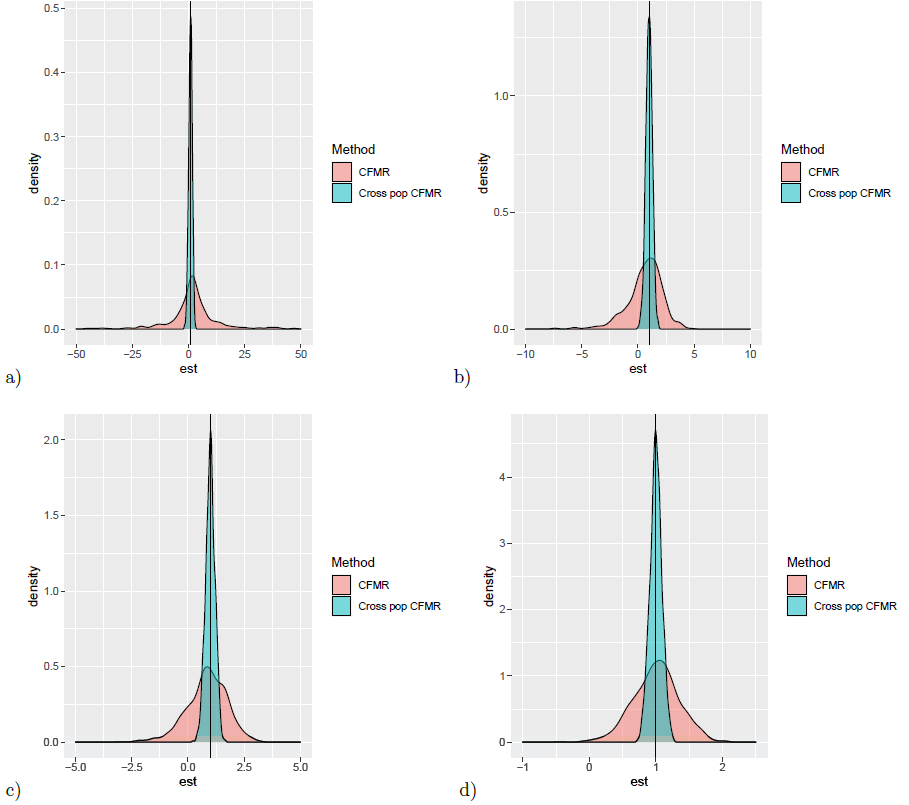

Supplement: S23 Fig — Panel a: the blue density represents the density of the cross-population CFMR (labeled as ‘cross pop CFMR’ in the figure) estimates of the parameter of interest in the simulation described in Section 3.3 for n = 1000. The vertical line corresponds to the true value of the parameter of interest, here equal to 1. The red density represents the density of the CFMR estimates of the parameter of interest in the simulation described in Section 3.3 for n = 1000. Panels b, c and d are the same as Panel a, except that n = 5000, 10,000 and 50,000 in each of these, respectively. (TIF) [file pcbi.1010268.s024.tif]
