# Supplementary material for: Cross-fitted instrument: A blueprint for one-sample Mendelian randomization
Source: PLoS Comput Biol. 2022 Aug 29;18(8):e1010268. doi: 10.1371/journal.pcbi.1010268 (PMC9462731; doi:10.1371/journal.pcbi.1010268)
Supplement: S1 Table — Estimated power of CFMR for different values of β0 and different sample sizes, with h2 = 20%. (PDF) [file pcbi.1010268.s025.pdf]

Sample size	β_0	Mean($\hat{\beta_0}$)	sd($\hat{\beta_0}$)	Mean($\widehat{sd(\hat{\beta_0})}$)	Power	Number of simulations [1]
1000	-0.080	-0.075	0.068	0.068	0.206	1000
1000	-0.050	-0.048	0.072	0.068	0.131	1000
1000	0.050	0.055	0.067	0.068	0.117	1000
1000	0.080	0.084	0.068	0.068	0.239	1000
2000	-0.080	-0.078	0.048	0.046	0.400	1000
2000	-0.050	-0.047	0.045	0.046	0.185	1000
2000	0.050	0.053	0.045	0.046	0.196	1000
2000	0.080	0.077	0.047	0.046	0.381	1000
3000	-0.080	-0.078	0.037	0.037	0.561	1000
3000	-0.050	-0.048	0.037	0.037	0.269	1000
3000	0.050	0.050	0.036	0.037	0.239	1000
3000	0.080	0.082	0.036	0.037	0.603	1000
4000	-0.080	-0.077	0.031	0.032	0.660	1000
4000	-0.050	-0.050	0.033	0.032	0.367	1000
4000	0.050	0.051	0.033	0.032	0.371	1000
4000	0.080	0.081	0.033	0.032	0.721	1000
5000	-0.080	-0.079	0.029	0.029	0.777	1000
5000	-0.050	-0.050	0.029	0.029	0.418	1000
5000	0.050	0.051	0.029	0.029	0.431	1000
5000	0.080	0.081	0.028	0.029	0.816	1000
6000	-0.080	-0.078	0.025	0.026	0.852	1000
6000	-0.050	-0.050	0.026	0.026	0.479	1000
6000	0.050	0.049	0.026	0.026	0.464	1000
6000	0.080	0.080	0.026	0.026	0.878	1000
7000	-0.080	-0.079	0.024	0.024	0.903	1000
7000	-0.050	-0.050	0.025	0.024	0.548	1000
7000	0.050	0.050	0.024	0.024	0.548	1000
7000	0.080	0.080	0.025	0.024	0.907	1000
8000	-0.080	-0.078	0.023	0.023	0.927	1000
8000	-0.050	-0.050	0.022	0.022	0.599	1000
8000	0.050	0.050	0.023	0.022	0.604	1000
8000	0.080	0.080	0.022	0.022	0.960	1000
9000	-0.080	-0.079	0.021	0.021	0.960	1000
9000	-0.050	-0.050	0.021	0.021	0.635	1000
9000	0.050	0.051	0.021	0.021	0.683	1000
9000	0.080	0.080	0.022	0.021	0.968	1000
10000	-0.080	-0.080	0.020	0.020	0.979	1000
10000	-0.050	-0.049	0.020	0.020	0.696	1000
10000	0.050	0.050	0.020	0.020	0.708	1000
10000	0.080	0.080	0.020	0.020	0.979	1000

[1] The column 'Sample size' corresponds to the sample size used in the simulation. 'β_0' is the effect of X on Y to be estimated. 'Mean $\hat{\beta_0}$' corresponds to the average estimate of β_0 across simulations. 'sd($\hat{\beta_0}$)' corresponds to the observed standard deviation of $\hat{\beta}$ across simulations. 'Mean ($\widehat{sd(\hat{\beta_0})}$)' corresponds to the average of the estimated standard deviation of $\hat{\beta_0}$. 'Power' corresponds to the proportion of the estimated P-value below 0.05. 'Number of simulations' is the number of simulations performed for the set of parameters (sample size, β_0, and h^2 in Table S**??**).
