# Supplementary material for: Cross-fitted instrument: A blueprint for one-sample Mendelian randomization
Source: PLoS Comput Biol. 2022 Aug 29;18(8):e1010268. doi: 10.1371/journal.pcbi.1010268 (PMC9462731; doi:10.1371/journal.pcbi.1010268)
Supplement: S2 Table — Type I error of CFMR for different sample sizes, with h2 = 20%. (PDF) [file pcbi.1010268.s026.pdf]

	α level			
Sample size	0.05	0.01	0.001	Number of simulations
1000	0.045	0.011	0.001	1000
2000	0.042	0.008	0.001	1000
3000	0.054	0.014	0.003	1000
4000	0.059	0.013	0.001	1000
5000	0.052	0.007	0.000	1000
6000	0.066	0.015	0.002	1000
7000	0.049	0.006	0.001	1000
8000	0.060	0.010	0.001	1000
9000	0.057	0.014	0.002	1000
10000	0.049	0.010	0.001	1000
