# Supplementary material for: Cross-fitted instrument: A blueprint for one-sample Mendelian randomization
Source: PLoS Comput Biol. 2022 Aug 29;18(8):e1010268. doi: 10.1371/journal.pcbi.1010268 (PMC9462731; doi:10.1371/journal.pcbi.1010268)
Supplement: S3 Table — Power of CFMR for different sample sizes and values of h2. (PDF) [file pcbi.1010268.s027.pdf]

h^2	Sample size	β_0	Mean $\hat{\beta}_0$	sd($\hat{\beta}_0$)	Mean($\hat{sd}(\hat{\beta}_0)$)	Power	Number of simulations
0.00000	1000	0.05	-0.1696	72.46	3997.26	0.019	1000
0.00000	1000	0.08	-0.1268	8.09	63.37	0.010	1000
0.00000	5000	0.05	-0.1581	126.03	23670.08	0.016	1000
0.00000	5000	0.08	-0.1396	16.03	260.51	0.007	1000
0.00000	10000	0.05	-0.1533	19.98	386.84	0.028	1000
0.00000	10000	0.08	-0.1061	15.26	226.39	0.006	1000
0.00000	50000	0.05	-0.1477	119.51	21979.87	0.012	335
0.00000	50000	0.08	-0.0712	106.42	5236.63	0.006	330
0.00000	100000	0.05	-0.1587	147.85	8001.65	0.012	335
0.00000	100000	0.08	-0.1087	5.55	36.49	0.015	330
0.00000	500000	0.05	0.0058	1.65	4.67	0.030	67
0.00000	500000	0.08	-0.0875	7.69	51.04	0.000	66
0.00001	100000	0.05	-0.1981	2.87	27.43	0.007	150
0.00001	100000	0.08	-0.2174	1.62	7.43	0.007	150
0.00010	100000	0.05	-0.2185	35.57	1316.74	0.013	150
0.00010	100000	0.08	-0.1148	2.22	5.30	0.013	150
0.00100	100000	0.05	0.0386	0.13	0.13	0.080	150
0.00100	100000	0.08	0.1057	0.13	0.13	0.140	150
0.01000	1000	0.05	-0.1168	10.55	113.30	0.010	1000
0.01000	1000	0.08	-0.1370	33.77	1006.66	0.014	1000
0.01000	5000	0.05	0.0520	21.79	196.09	0.020	1000
0.01000	5000	0.08	0.0852	65.17	2489.32	0.035	1000
0.01000	10000	0.05	0.0629	0.13	0.13	0.057	1000
0.01000	10000	0.08	0.0961	0.13	0.13	0.087	1000
0.01000	50000	0.05	0.0501	0.04	0.05	0.158	330
0.01000	50000	0.08	0.0730	0.05	0.05	0.385	335
0.01000	100000	0.05	0.0550	0.03	0.03	0.408	480
0.01000	100000	0.08	0.0802	0.03	0.03	0.707	485
0.01000	500000	0.05	0.0499	0.01	0.01	0.939	198
0.01000	500000	0.08	0.0817	0.01	0.01	1.000	201
0.05000	1000	0.05	0.0503	5.09	16.17	0.026	1000
0.05000	1000	0.08	0.0840	6.92	34.68	0.018	1000
0.05000	5000	0.05	0.0539	0.06	0.07	0.117	1000
0.05000	5000	0.08	0.0847	0.07	0.07	0.243	1000
0.05000	10000	0.05	0.0513	0.04	0.04	0.184	1000
0.05000	10000	0.08	0.0818	0.04	0.04	0.453	1000
0.05000	50000	0.05	0.0514	0.02	0.02	0.749	335
0.05000	50000	0.08	0.0800	0.02	0.02	0.979	335
0.05000	100000	0.05	0.0529	0.01	0.01	0.958	335
0.05000	100000	0.08	0.0776	0.01	0.01	1.000	335
0.05000	500000	0.05	0.0495	0.01	0.01	1.000	67
0.05000	500000	0.08	0.0794	0.01	0.01	1.000	67
0.10000	1000	0.05	0.0497	0.12	0.12	0.047	1000
0.10000	1000	0.08	0.0817	0.12	0.12	0.095	1000
0.10000	5000	0.05	0.0486	0.04	0.04	0.210	1000
0.10000	5000	0.08	0.0838	0.05	0.04	0.485	1000
0.10000	10000	0.05	0.0515	0.03	0.03	0.374	1000
0.10000	10000	0.08	0.0799	0.03	0.03	0.760	1000
0.10000	50000	0.05	0.0508	0.01	0.01	0.958	335
0.10000	50000	0.08	0.0803	0.01	0.01	1.000	330
0.10000	100000	0.05	0.0509	0.01	0.01	1.000	335
0.10000	100000	0.08	0.0796	0.01	0.01	1.000	330
0.10000	500000	0.05	0.0500	0.00	0.00	1.000	201
0.10000	500000	0.08	0.0801	0.00	0.00	1.000	198
0.20000	1000	0.05	0.0527	0.07	0.07	0.114	1000
0.20000	1000	0.08	0.0816	0.07	0.07	0.233	1000

Table – *Continued from previous page*

h^2	Sample size	β_0	Mean $\hat{\beta}_0$	sd($\hat{\beta}_0$)	Mean($\hat{sd}(\hat{\beta}_0)$)	Power	Number of simulations
0.20000	5000	0.05	0.0505	0.03	0.03	0.420	1000
0.20000	5000	0.08	0.0799	0.03	0.03	0.809	1000
0.20000	10000	0.05	0.0499	0.02	0.02	0.705	1000
0.20000	10000	0.08	0.0805	0.02	0.02	0.981	1000
0.20000	50000	0.05	0.0495	0.01	0.01	1.000	330
0.20000	50000	0.08	0.0798	0.01	0.01	1.000	335
0.20000	100000	0.05	0.0505	0.01	0.01	1.000	330
0.20000	100000	0.08	0.0803	0.01	0.01	1.000	335
0.20000	500000	0.05	0.0497	0.00	0.00	1.000	132
0.20000	500000	0.08	0.0798	0.00	0.00	1.000	134
