# Supplementary material for: Cross-fitted instrument: A blueprint for one-sample Mendelian randomization
Source: PLoS Comput Biol. 2022 Aug 29;18(8):e1010268. doi: 10.1371/journal.pcbi.1010268 (PMC9462731; doi:10.1371/journal.pcbi.1010268)
Supplement: S4 Table — Type I error of CFMR for different sample sizes and values of h2. (PDF) [file pcbi.1010268.s028.pdf]

		α level			
h^2	Sample size	0.05	0.01	0.001	Number of simulations
0.00000	1000	0.0380	0.0000	0.0000	1000
0.00000	5000	0.0240	0.0010	0.0000	1000
0.00000	10000	0.0270	0.0050	0.0010	1000
0.00000	50000	0.0239	0.0090	0.0000	335
0.00000	100000	0.0209	0.0000	0.0000	335
0.00000	500000	0.0299	0.0000	0.0000	67
0.00001	1000	0.0260	0.0020	0.0000	1000
0.00001	5000	0.0210	0.0030	0.0000	1000
0.00001	10000	0.0280	0.0020	0.0000	1000
0.00001	50000	0.0250	0.0040	0.0000	1000
0.00001	100000	0.0333	0.0067	0.0000	150
0.00010	1000	0.0270	0.0040	0.0000	1000
0.00010	5000	0.0250	0.0010	0.0000	1000
0.00010	10000	0.0290	0.0040	0.0000	1000
0.00010	50000	0.0200	0.0020	0.0000	1000
0.00010	100000	0.0333	0.0000	0.0000	150
0.00100	1000	0.0270	0.0010	0.0000	1000
0.00100	5000	0.0250	0.0020	0.0000	1000
0.00100	10000	0.0300	0.0040	0.0000	1000
0.00100	50000	0.0230	0.0010	0.0000	1000
0.00100	100000	0.0800	0.0200	0.0000	150
0.01000	1000	0.0240	0.0020	0.0000	2000
0.01000	5000	0.0230	0.0030	0.0005	2000
0.01000	10000	0.0445	0.0075	0.0000	2000
0.01000	50000	0.0487	0.0067	0.0007	1335
0.01000	100000	0.0722	0.0206	0.0021	485
0.01000	500000	0.0597	0.0100	0.0000	201
0.05000	1000	0.0260	0.0040	0.0000	1000
0.05000	5000	0.0430	0.0110	0.0020	1000
0.05000	10000	0.0480	0.0080	0.0010	1000
0.05000	50000	0.0273	0.0061	0.0000	330
0.05000	100000	0.0697	0.0182	0.0000	330
0.05000	500000	0.0758	0.0000	0.0000	66
0.10000	1000	0.0560	0.0050	0.0000	1000
0.10000	5000	0.0410	0.0080	0.0010	1000
0.10000	10000	0.0470	0.0140	0.0010	1000
0.10000	50000	0.0388	0.0060	0.0000	335
0.10000	100000	0.0537	0.0119	0.0000	335
0.10000	500000	0.0398	0.0000	0.0000	201
0.20000	1000	0.0400	0.0100	0.0010	1000
0.20000	5000	0.0510	0.0120	0.0010	1000
0.20000	10000	0.0490	0.0120	0.0030	1000
0.20000	50000	0.0657	0.0119	0.0000	335
0.20000	100000	0.0657	0.0090	0.0000	335
0.20000	500000	0.0522	0.0075	0.0000	134
