# Supplementary material for: Cross-fitted instrument: A blueprint for one-sample Mendelian randomization
Source: PLoS Comput Biol. 2022 Aug 29;18(8):e1010268. doi: 10.1371/journal.pcbi.1010268 (PMC9462731; doi:10.1371/journal.pcbi.1010268)
Supplement: S5 Table — (PDF) [file pcbi.1010268.s029.pdf]

$-log_{10}$ SNP P-value	Variance explained by CFI	CFMR estimate	Std. Error error	P-value	95% CI lower limit	95% CI upper limit
-3	1.112 %	24.130	5.938	0.00005	12.491	35.768
-4	1.102 %	27.031	6.257	0.00002	14.767	39.295
-5	1.101 %	22.399	6.308	0.00038	10.035	34.762
-6	1.112 %	19.571	5.910	0.00093	7.986	31.155
-7	0.951 %	17.420	6.409	0.00657	4.859	29.980
-8	0.044 %	20.669	9.125	0.02351	2.785	38.553
