# Supplementary material for: Cross-fitted instrument: A blueprint for one-sample Mendelian randomization
Source: PLoS Comput Biol. 2022 Aug 29;18(8):e1010268. doi: 10.1371/journal.pcbi.1010268 (PMC9462731; doi:10.1371/journal.pcbi.1010268)
Supplement: S6 Table — (PDF) [file pcbi.1010268.s030.pdf]

$-log_{10}$ SNP P-value	Confounder	Estimate	Std. Error	P-value
-3	Mother's age	-0.012	0.015	0.4254063
-3	Mother's education	-0.014	0.007	0.0548912
-3	Gestational age	0.017	0.005	0.0014255
-3	Mother's smoking	0.033	0.008	0.0000443
-4	Mother's age	-0.004	0.020	0.8356098
-4	Mother's education	-0.005	0.009	0.6290257
-4	Gestational age	0.027	0.007	0.0001246
-4	Mother's smoking	0.039	0.011	0.0002364
-5	Mother's age	-0.029	0.033	0.3819641
-5	Mother's education	0.008	0.016	0.6245407
-5	Gestational Age	0.051	0.012	0.0000125
-5	Mother's smoking	0.089	0.018	0.0000005
-6	Mother's age	-0.087	0.066	0.1878742
-6	Mother's education	0.003	0.031	0.9251442
-6	Gestational Age	0.068	0.023	0.0034211
-6	Mother's smoking	0.121	0.035	0.0005351
-7	Mother's age	-0.105	0.131	0.4222781
-7	Mother's education	0.027	0.061	0.6548937
-7	Gestational Age	0.075	0.046	0.1035003
-7	Mother's smoking	0.003	0.069	0.9634737
-8	Mother's age	-0.372	0.227	0.1006788
-8	Mother's education	-0.052	0.106	0.6247780
-8	Gestational age	0.088	0.079	0.2663506
-8	Mother's smoking	-0.001	0.120	0.9932419
