# Supplementary material for: Cross-fitted instrument: A blueprint for one-sample Mendelian randomization
Source: PLoS Comput Biol. 2022 Aug 29;18(8):e1010268. doi: 10.1371/journal.pcbi.1010268 (PMC9462731; doi:10.1371/journal.pcbi.1010268)
Supplement: S7 Table — Estimation of the effect of X on Y for β = 0.8 by one-sample MR and CFMR, respectively. The simulations are detailed in Section 3.2. (PDF) [file pcbi.1010268.s031.pdf]

N	h^2	CFMR $\hat{\beta}_0$ estimate	1SMR $\hat{\beta}$ estimate	MR RAPS $\hat{\beta}$ estimate	Barry *et al.* $\hat{\beta}$ estimate
1,000	0.10	0.67	4.65	17.94	11.20
5,000	0.10	0.71	1.51	5.26	4.08
10,000	0.10	0.80	1.18	2.81	2.55
50,000	0.10	0.79	0.87	1.17	1.17
1,000	0.20	0.64	2.47	11.80	7.18
5,000	0.20	0.77	1.11	2.58	2.40
10,000	0.20	0.79	0.95	1.65	1.63
50,000	0.20	0.80	0.83	0.97	0.97
