# Supplementary material for: Cross-fitted instrument: A blueprint for one-sample Mendelian randomization
Source: PLoS Comput Biol. 2022 Aug 29;18(8):e1010268. doi: 10.1371/journal.pcbi.1010268 (PMC9462731; doi:10.1371/journal.pcbi.1010268)
Supplement: S8 Table — Estimation of the effect of X on Y for β = 1 by cross-population CFMR (cpCFMR) and CFMR, respectively. The simulations are detailed in Section 3.3. Each scenario has been simulated 1000 times. (PDF) [file pcbi.1010268.s032.pdf]

N	Mean($\hat{\beta}_0^{cp*}$)	sd($\hat{\beta}_0^{cp}$)	Mean(sd($\hat{\beta}_0^{cp}$))	Mean ($\hat{\beta}_0^{**}$)	sd($\hat{\beta}_0$)	Mean(sd($\hat{\beta}_0$))
1,000	1.04	0.78	0.71	0.53	28.65	28.65
5,000	0.99	0.27	0.29	0.54	1.57	1.57
10,000	1.00	0.18	0.20	0.89	0.90	0.90
50,000	1.00	0.09	0.09	1.00	0.34	0.34

$*$: $\hat{\beta}_0^{cp}$= cross-population CFMR

$**$: $\hat{\beta}_0$= CFMR
